# Regulatory roles of IL-10–producing human follicular T cells

**DOI:** 10.1084/jem.20190493

**Published:** 2019-06-17

**Authors:** Pablo F. Cañete, Rebecca A. Sweet, Paula Gonzalez-Figueroa, Ilenia Papa, Naganari Ohkura, Holly Bolton, Jonathan A. Roco, Marta Cuenca, Katharine J. Bassett, Ismail Sayin, Emma Barry, Angel Lopez, David H. Canaday, Michael Meyer-Hermann, Claudio Doglioni, Barbara Fazekas de St Groth, Shimon Sakaguchi, Matthew C. Cook, Carola G. Vinuesa

**Affiliations:** 1 Department of Immunology and Infectious Disease and Centre for Personalised Immunology, The John Curtin School of Medical Research, The Australian National University, Canberra, Australia; 2 Department of Immunology, Canberra Hospital, Canberra, Australia; 3 Laboratory of Experimental Immunology, World Premier International Research Center Immunology Frontier Research Center, Osaka University, Osaka, Japan; 4 Discipline of Pathology, School of Medical Sciences, Charles Perkins Centre, University of Sydney, New South Wales, Australia; 5 Division of Infectious Diseases and HIV Medicine, Case Western Reserve University, Cleveland, OH; 6 Cytokine Receptor Laboratory, Centre for Cancer Biology, Adelaide, Australia; 7 Department of Systems Immunology and Braunschweig Integrated Centre of Systems Biology, Helmholtz Centre for Infection Research, Braunschweig, Germany; 8 Department Pathology, San Raffaele Scientific Institute, Università Vita-Salute, Milan, Italy

## Abstract

Mucosal lymphoid tissues such as human tonsil are colonized by bacteria and exposed to ingested and inhaled antigens, requiring tight regulation of immune responses. Antibody responses are regulated by follicular helper T (T_FH_) cells and FOXP3^+^ follicular regulatory T (T_FR_) cells. Here we describe a subset of human tonsillar follicular T cells identified by expression of T_FH_ markers and CD25 that are the main source of follicular T (T_F_) cell–derived IL-10. Despite lack of FOXP3 expression, CD25^+^ T_F_ cells resemble T reg cells in high CTLA4 expression, low IL-2 production, and their ability to repress T cell proliferation. CD25^+^ T_F_ cell–derived IL-10 dampens induction of B cell class-switching to IgE. In children, circulating total IgE titers were inversely correlated with the frequencies of tonsil CD25^+^ T_F_ cells and IL-10–producing T_F_ cells but not with total T reg cells, T_FR_, or IL-10–producing T cells. Thus, CD25^+^ T_F_ cells emerge as a subset with unique T and B cell regulatory activities that may help prevent atopy.

## Abstract (precis)

Uncontrolled IgE responses drive allergies and anaphylaxis. Here, Cañete et al. describe a human follicular regulatory T cell population that does not express FOXP3 and produces abundant IL-10, which limits IgE switching. These cells appear to be key regulators of atopy.

## Introduction

High-affinity antibodies are critical for long-lived host defense after infection or vaccination. Conversely, dysregulation of antibody responses is the basis of both serious autoimmune diseases and allergy (Vinuesa et al., 2016). It is clear that specialized B cell lymphoma 6 protein (BCL6)–driven B helper follicular T (T_FH_) cells are essential in supporting and regulating the quality and longevity of antibody responses (Crotty, 2011; Vinuesa et al., 2016). T_FH_ cells first interact with antigen-specific B cells at the borders between T cell zones and B cell follicles, driving B cells to differentiate in extrafollicular foci as short-lived plasmablasts (Lee et al., 2011), and then after repeated cycles of division and mutation within germinal centers (GCs). T_FH_ cells also drive GC B cell differentiation into long-lived plasma cells and memory B cells. Limiting T_FH_ cell help appears to be crucial for long-lived high-affinity antibody responses (Victora et al., 2010), and aberrant accumulation of T_FH_ cells has been shown to promote selection of GC B cells and lead to autoantibodies (Vinuesa et al., 2005; Linterman et al., 2009; Simpson et al., 2010) and IgE^+^ B cells (Yang et al., 2016). The BCL6^+^ follicular T (T_F_) cell population also contains regulatory cells made up of a thymic-derived and peripherally induced forkhead box P3 (FOXP3)–expressing population known as follicular regulatory T cells (T_FR_; Chung et al., 2011; Linterman et al., 2011; Wollenberg et al., 2011). In mice, T_FR_ cells have been shown to repress GC B cells and antibody responses (Sage et al., 2016). Regulatory CD25^+^ T cells and follicular FOXP3^+^ T cells have also been reported in humans (Lim et al., 2004; Carreras et al., 2006; Chung et al., 2011) and circulating follicular FOXP3^+^ regulatory populations have been described (Fonseca et al., 2017; Wing et al., 2017). Nevertheless, the nature of T_FR_ cells in human tonsil, the most accessible human secondary lymphoid tissue, remains uncharacterized. CD25^+^ T_F_ cells have been reported in human tonsils but are not considered to carry out regulatory roles based on their lack of FOXP3 expression (Li and Pauza, 2015), even though functional studies are lacking.

Anaphylaxis and other acute allergic reactions are growing in incidence and are poorly understood problems causing increasing morbidity and mortality (Yue et al., 2018). These reactions are driven by cross-linking of IgE bound to the high-affinity IgE receptor (FcεRI) on mast cells and basophils, which leads to the release of inflammatory and vasoactive mediators (Galli et al., 2008). Allergic pathology is often located at epithelial and mucosal sites and consists of type 2 immune responses, in which signature cytokines IL-4 and IL-13 are derived from type 2 innate lymphoid (ILC2) cells, basophils, or CD4^+^ helper T cells (Voehringer et al., 2006; Licona-Limón et al., 2013; Hammad and Lambrecht, 2015). These signature cytokines drive B cells to undergo class switch recombination (CSR) to IgE. There is evidence that IgE-producing plasma cells can arise both upon T cell priming of B cell differentiation along the extrafollicular route and upon interaction with T cells within epithelial lesions as a result of sequential CSR in IgG memory B cells that arose in GCs (Erazo et al., 2007; Xiong et al., 2012; He et al., 2013). IgE^+^ B cells can also be found in GCs (He et al., 2013), and several lines of evidence have suggested that T_FH_ cells contribute to IgE production (Glatman Zaretsky et al., 2009; King and Mohrs, 2009; Reinhardt et al., 2009; Coquet et al., 2015; Ballesteros-Tato et al., 2016). Recently, a dependence of T_FH_ cells has been confirmed for mouse IgE responses induced by airborne antigens (Kobayashi et al., 2017). IgE responses appear to be tightly regulated, particularly in the context of GC responses, with several B cell–intrinsic mechanisms shown to limit IgE production (Yang et al., 2012; He et al., 2013; Butt et al., 2015). To date, despite an established role of regulatory T (T reg) cells in IgE repression (Josefowicz et al., 2012a), whether T_FR_ cells contribute to IgE regulation remains unclear.

Here, we describe unique regulatory functions of human tonsillar follicular CD25^+^ T cells, which are the main producers of IL-10 among human T_F_ cells. Despite exhibiting similarities to mouse T_FR_ cells both phenotypically and functionally, CD25^+^ T_F_ cells in tonsil are FOXP3 negative. Strikingly, these cells exert a strong suppression on human T_FH_ cells and expression of key molecules BCL6, CD40L, and IL-21, while not directly suppressing plasma cell differentiation of B cells. CD25^+^ T_F_ cell–derived IL-10 dampened induction of Ig switching to IgE. In children, serum total IgE titers were inversely correlated with the frequencies of tonsil CD25^+^ T_F_ cells and IL-10–producing T_F_ cells, but not with total T reg cells, T_FR_ cells, or IL-10–producing T cells. Thus, CD25^+^ T_F_ cells emerge as a subset with unique T and B cell regulatory activities that may help prevent atopy.

## Results

### Identification of tonsillar CD25^+^ FOXP3^−^ T_F_ cells that express abundant IL-10

**Figure 1.**
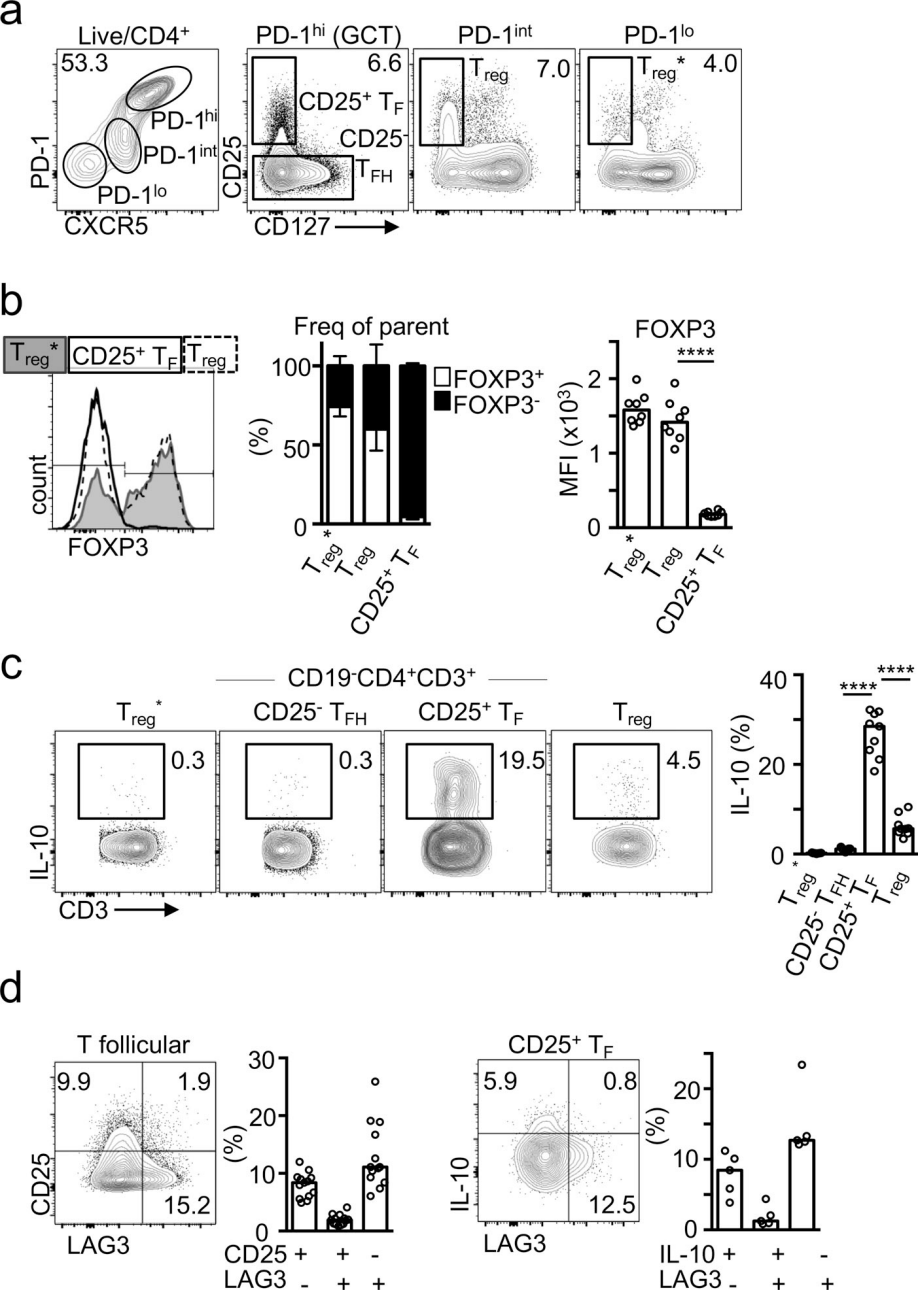
**Identification of IL-10–producing CD25^+^ FOXP3^−^ human T_F_ cells in human tonsils. (a)** Flow cytometric plots showing gating strategy to identify the indicated populations. **(b)** Flow cytometric plots and quantification (*n* = 8) showing percentage of FOXP3^+^ cells and FOXP3 mean fluorescence intensity (MFI) within the indicated subset according to panel a. Data are representative of 10 independent experiments. **(c)** Flow cytometric plots and quantification of PMA/ionomycin-stimulated tonsillar cell suspensions showing IL-10 expression in the indicated subset (*n* = 9). Data are representative of 10 experiments. **(d)** Flow cytometric plots and quantification showing LAG3 and CD25 expression in total T_F_ cells (*n* = 14; left), and IL-10 and LAG3 in CD25^+^ T_F_ cells (*n* = 5; right). Data are representative of five independent experiments. In all graphs, bars represent medians, and each dot represents a single tonsil donor. ****, P ≤ 0.0001, nonparametric Mann–Whitney *U* test.

In an effort to identify the human equivalent of mouse T_FR_ cells, we stained cells from the most accessible human secondary lymphoid tissue, tonsil, for markers of T_F_ cells and T reg cells. Total T reg cells were identified by expression of CD25 in the absence of CD127 (Seddiki et al., 2006; Fig. 1 a). The majority of CD25^+^ CD127^−^ T reg cells found within the nonfollicular T effector gate (C-X-C motif chemokine receptor 5 [CXCR5]^int^ and programmed cell death protein 1 [PD-1]^int^) expressed FOXP3, and as such constitute the conventional T reg cell population (Fig. 1, a and b). A CD25^+^ CD127^−^ subset was also identified within the follicular CXCR5^hi^ PD-1^hi^ population, which we refer to as “CD25^+^ T_F_” cells (Fig. 1 a). We speculated that these might correspond to the T_FR_ cell population identified in mice. To our surprise, the majority (∼95%) of these tonsillar CD25^+^ CD127^−^ T_F_ cells lacked FOXP3 expression (P < 0.0001; Fig. 1 b). We also identified follicular CD25^+^ FOXP3^+^ and CD25^−^ FOXP3^+^ T cells (Fig. S3 a). CD25^+^ FOXP3^−^ T_F_ cells were also present in human mesenteric lymph nodes, although these were less frequent than those seen in the tonsil and found at proportions comparable to CD25^+^ FOXP3^+^ T cells (P = 0.2236; Fig. S1).

We were intrigued by the lack of FOXP3 expression among the majority of CD25^+^ T_F_ cells and considered that CD25 expression may be either identifying activated T_FH_ cells or a T reg cell subset independent of FOXP3 expression. FOXP3-negative T reg cells have been described in humans and mice, and among these are type 1 regulatory (Tr1) cells that produce IL-10 and are identified by expression of the lymphocyte activating gene 3 (LAG3; Gagliani et al., 2013). We therefore investigated the relative ability of the different tonsil T cell subsets to produce IL-10. Strikingly, staining for IL-10 revealed that the FOXP3^−^ CD25^+^ T_F_ cell population was the subset containing the largest fraction of IL-10–producing T cells in human tonsil (Fig. 1 c): 20–30% expressed IL-10 compared with 3–12% of conventional T reg cells (P < 0.0001) and barely any T_FH_ cells (P < 0.0001; Fig. 1 c). Unlike IL-10–producing Tr1 cells, IL-10–producing T_F_ cells did not express LAG3 (Fig. 1 d), suggesting that CD25^+^ T_F_ cells may not be Tr1 cells with a follicular phenotype.

**Figure 2.**
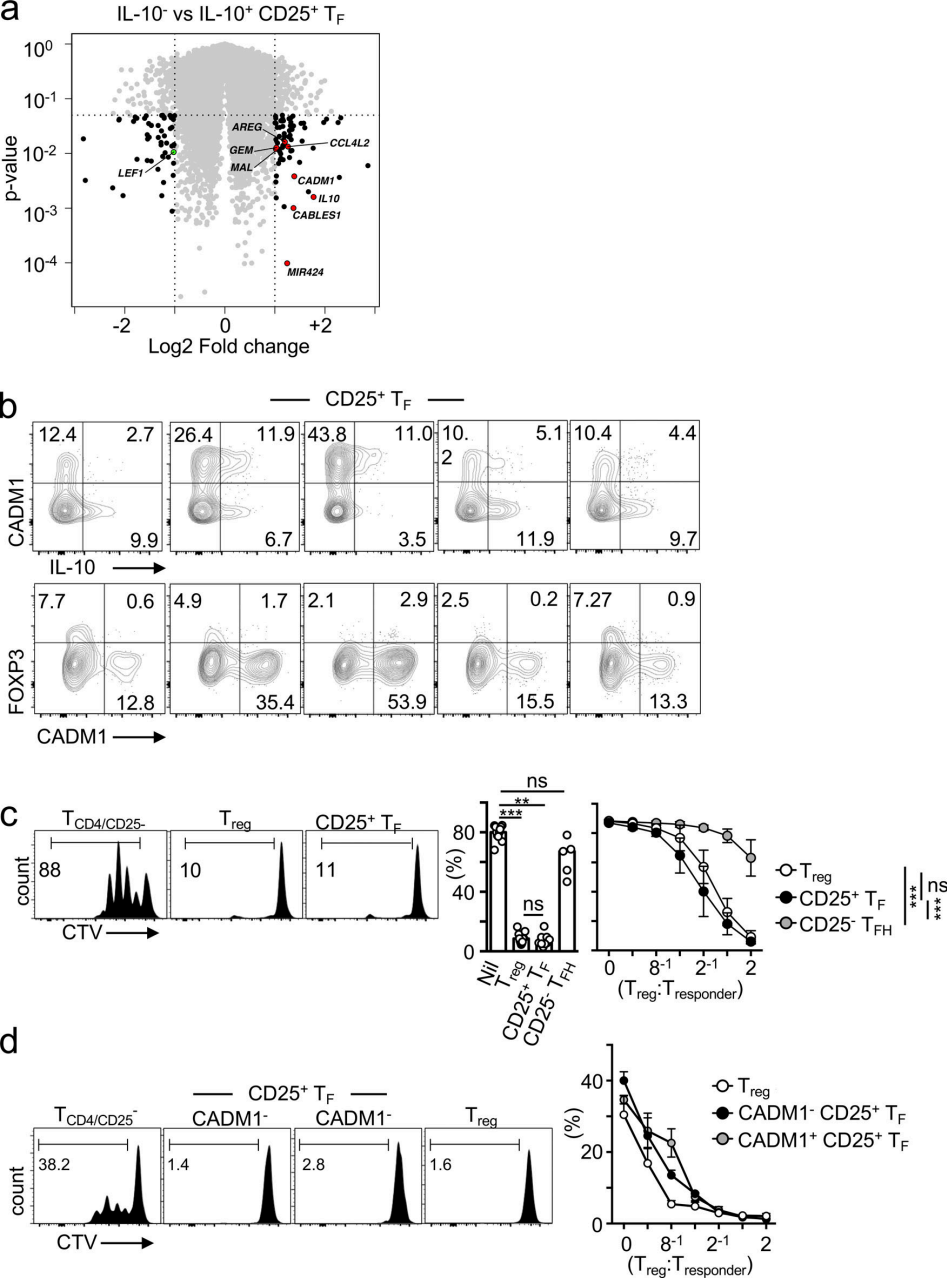
**Human CD25^+^ T_F_ cells repress T cell proliferation. (a)** Volcano plot showing Affymetrix RNA microarrays comparing gene expression between IL-10–producing and nonproducing T_F_ cells (*n* = 3). **(b)** Flow cytometric plots showing coexpression of CADM1 with IL-10 or FOXP3 in PMA/ionomycin-stimulated tonsillar cell suspensions in the indicated subset (*n* = 5). Data are representative of two independent experiments. **(c and d)** Flow cytometric plots and quantification of proliferating CD4^+^CD25^−^ responder T cells according to dilution of the cytoplasmic fluorescent dye CTV after 3 d of α-CD3 and α-CD28 stimulation in the presence or absence of T reg cells (*n* = 10), CD25^+^ T_F_ cells (*n* = 8), and CD25^−^ T_FH_ cells (*n* = 5; c) or T reg cells (*n* = 3), CADM1^+^CD25^+^ T_F_ cells (*n* = 3), and CADM1^−^CD25^−^ T cells (*n* = 3; d). Bars represent medians, and each dot represents the mean value of cultures set up in triplicate from a single donor. Data are representative of five (c) and two (b and d) independent experiments. ns, not significant; **, P ≤ 0.01; ***, P ≤ 0.001, nonparametric Mann–Whitney *U* test (c, left panel) and two-way ANOVA (c, right panel, and d).

Having demonstrated that only a fraction of CD25^+^ T_F_ cells expressed IL-10, we asked whether IL-10^+^ and IL-10^−^ T_F_ cells were fundamentally different subsets. Transcriptional profiling using Affymetrix RNA microarrays of IL-10–producing versus nonproducing T_F_ cells revealed highly comparable transcriptomes (Fig. 2 a), with only a few differentially expressed transcripts including *IL-10*, C-C motif chemokine 4-like (*CCL4L2*), T-lymphocyte maturation-associated protein (*MAL*), *microRNA424* (*MIR424*; reported to be an activator of TGF-β signaling; Li et al., 2014), lymphoid enhancer binding factor 1 (*LEF1*; a transcription factor important for T_FH_ differentiation; Choi et al., 2015), and the surface receptor cell adhesion molecule 1 (*CADM1*), which has been previously reported to mark a population of adult T cell leukemia/lymphoma (Komohara et al., 2017). To evaluate whether CADM1 expression could identify the IL-10–producing CD25^+^ T_F_ cells, we stained human tonsils with an anti-CADM1 antibody together with T_F_- and T reg cell–specific markers. While CADM1 expression failed to detect all IL-10–producing cells within the CD25^+^ T_F_ population (Fig. 2 b, upper panels), it did exclude most FOXP3-expressing T_F_ cells (Fig. 2 b, lower panels). Altogether, these results suggest that IL-10–producing and nonproducing CD25^+^ T_F_ cells are closely related, with IL-10 probably being produced upon CD25^+^ T_F_ cell activation.

### CD25^+^ T_F_ cells suppress T cell proliferation

To investigate whether CD25^+^ T_F_ cells were simply activated T_FH_ cells that had up-regulated CD25, as previously proposed (Li and Pauza, 2015) and as opposed to T reg cells, we tested their ability to suppress T cell proliferation in vitro. Unlike typical T_FH_ cells (CD25^−^ CD127^−^), human CD25^+^ T_F_ cells and T reg cells were equally effective in suppressing conventional (CD25^−^ CD4^+^) T cell proliferation (Fig. 2 c), as previously described for mouse T_FR_ cells (Chung et al., 2011; Linterman et al., 2011). While it is unlikely that the small contaminating number of FOXP3^+^ cells among tonsillar CD25^+^ T_F_ cells (i.e., the equivalent to mouse T_FR_ cells) is responsible for the overall suppressive effect of this population in the cell titration response, we sought to exclude the contribution from such T_FR_ cells. We used CADM1 to sort FOXP3^−^ CD25^+^ T_F_ cells. CADM1^+^ and CADM1^−^ T_F_ cells suppressed T cell proliferation as effectively as T reg cells (Fig. 2 d), demonstrating that CD25^+^ T_F_ cells exert T reg cell functions even in the absence of FOXP3 expression. Given that CADM1 does not distinguish IL-10^+^ versus IL-10^−^ CD25 T_F_ cells, it is unlikely that IL-10 mediates suppression of T cell proliferation. Indeed, IL-10 blockade in these cultures did not affect this regulatory property of CD25^+^ T_F_ cells (data not shown). Altogether, these results suggest that human CD25^+^ CD127^−^ T_F_ cells are not simply activated T_FH_ cells, but can behave like T reg cells.

**Figure 3.**
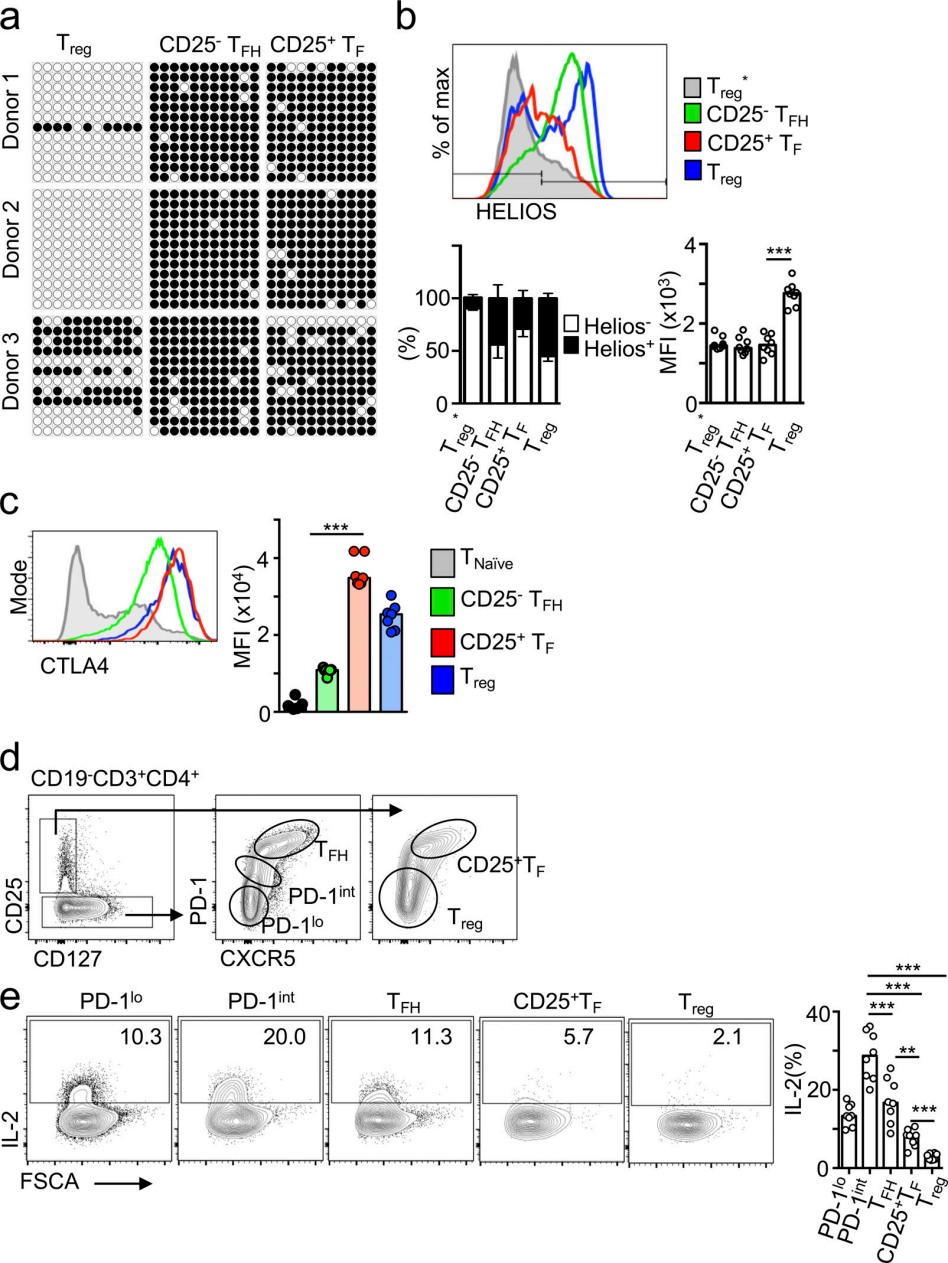
**Human CD25^+^ T_F_ cells may be peripherally induced and express high CTLA4 but low IL-2 production. (a)** Bisulfite sequencing of the 11 CpG islands of the *FOXP3* CNS2 locus with 12 representative clones per population and per donor (filled circle, methylated; open circle, demethylated). **(b)** Flow cytometric plots and quantification showing HELIOS expression in the indicated cell subsets (*n* = 8). Data are representative of three independent experiments. **(c)** Flow cytometric plots and quantification (*n* = 8) showing CTLA4 expression in the indicated cell subset. Data are representative of five independent experiments. **(d and e)** Flow cytometric plots and quantification showing gating strategy (d) and IL-2 expression of PMA/ionomycin-stimulated tonsillar cell suspensions in the indicated subset (*n* = 8; e). Data are representative of two independent experiments. In all graphs, bars represent medians, and each dot represents a single donor. **, P ≤ 0.01; ***, P ≤ 0.001, nonparametric Mann–Whitney *U* test. FSCA, forward scatter.

To gain insights into the ontogeny of CD25^+^ T_F_ cells, and specifically ask whether these cells may have expressed FOXP3 as thymic T reg cells (tT reg cells) and later down-regulated it, we investigated the methylation of the conserved noncoding sequence 2 (CNS2) locus of the *FOXP3* promoter. Demethylation of this locus is critical for the stable FOXP3 expression that characterizes tT reg cells; Zheng et al., 2010). The CNS2 locus was >90% methylated in human CD25^+^ T_F_ cells (Fig. 3 a), suggesting a peripheral rather than thymic origin. Similarly, compared with conventional nonfollicular T reg cells, CD25^+^ T_F_ cells expressed 40% less HELIOS (Fig. 3 b, P = 0.0002), a known target of FOXP3 (Fu et al., 2012) that is abundant in, although not exclusive of, tT reg cells (Thornton et al., 2010; Szurek et al., 2015). Together, these data demonstrate the existence of human CD25^+^ FOXP3^−^ T_FR_ cells that are unlikely to originate from tT reg cells and appear to be enriched in tonsillar tissue.

We next investigated whether, in spite of absent FOXP3 expression, CD25^+^ T_F_ cells display the two key characteristics of conventional T reg cells known to be crucial for their suppressor function: high cytotoxic T lymphocyte–associated protein 4 (CTLA4) expression and low IL-2 production. CTLA4 is one of the most important targets of FOXP3 in T reg cells, and high amounts of CTLA4 expression plus absence of IL-2 production is a good marker of T reg cell function even in the absence of FOXP3 expression (Yamaguchi et al., 2013). We found that CD25^+^ T_F_ cells had the highest amount of CTLA4 among tonsil T cell subsets (Fig. 3 c), and IL-2 production was detected in only ∼5% of human tonsil CD25^+^ T_F_ cells (Fig. 3, d and e). These results further suggest that CD25^+^ T_F_ cells are indeed a T reg cell subset.

### Human CD25^+^ T_F_ cells resemble mouse T_FR_ cells

**Figure 4.**
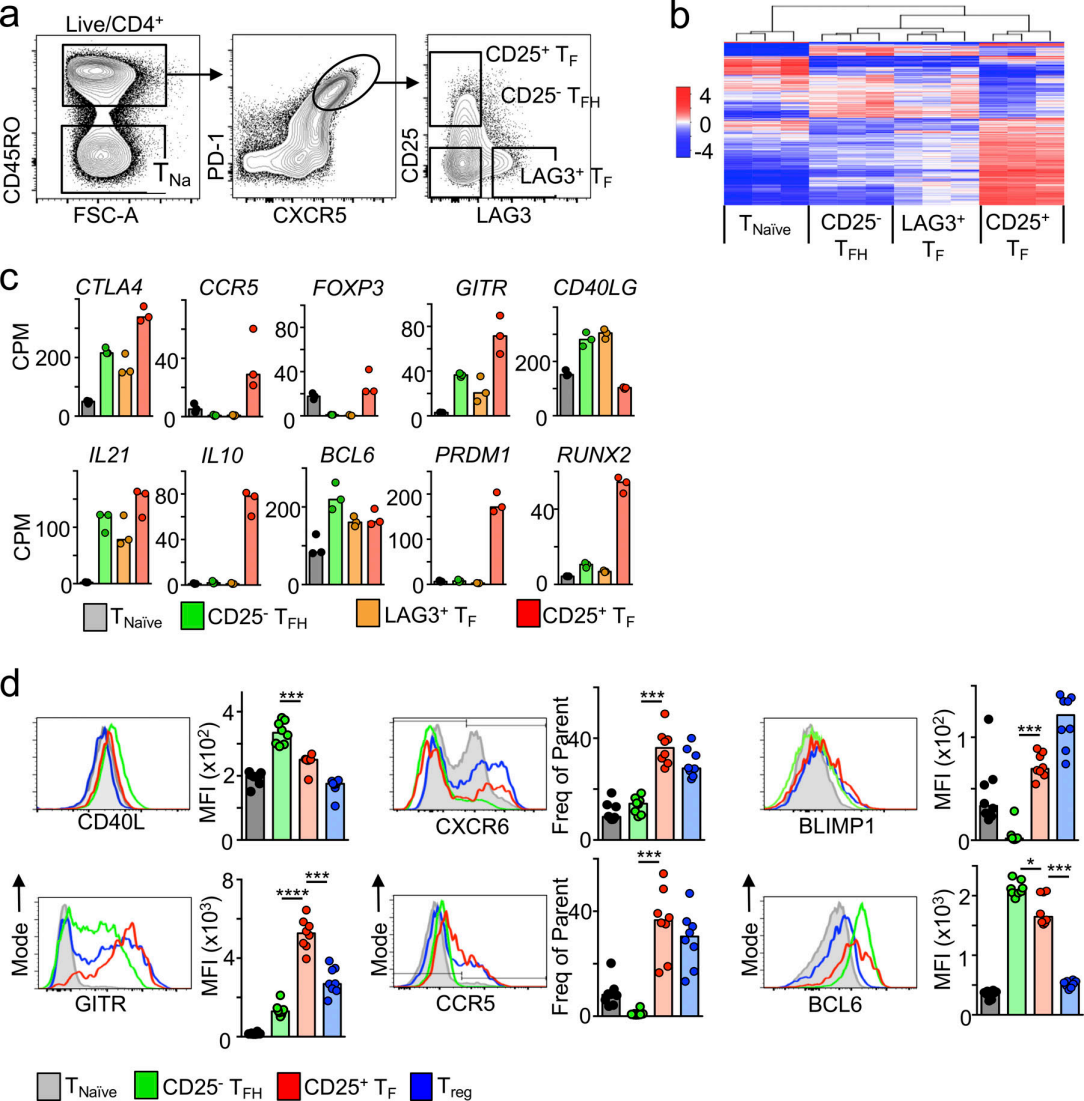
**Human CD25^+^ T_F_ cells’ transcriptome resembles that of murine T_FR_ cells. (a)** Flow cytometric plots showing the gating strategy used to FACS-purify each of the indicated cell subsets. **(b)** Heatmap analysis of RNA-seq data showing differentially expressed genes in CD25^+^ T_F_ cells compared with the indicated T cell populations (log_2_ value of counts per million) extracted from the tonsils of three individuals. **(c)** Selected transcripts from panel b in the indicated subsets (RNA counts per million [CPM]). **(d)** Flow cytometric plots and quantification (*n* = 8) of the indicated proteins. Data are representative of at least three independent experiments. In all graphs, bars represent medians; each dot represents a single tonsil donor. ***, P ≤ 0.001, nonparametric Mann–Whitney *U* test.

To investigate the extent to which human CD25^+^ T_F_ cells resemble mouse T_FR_ cells, we obtained the transcriptional signature of CD25^+^ T_F_ cells. As shown before, we noted that among T_F_ cells, CD25^+^ T_F_ cells did not overlap with LAG3^+^ T_F_ cells. Thus, we FACS-purified naive T cells and the three major T_F_ cell subsets according to CD25 and LAG3 expression, CD25^+^ LAG3^−^ T_F_ cells, CD25^−^ LAG3^+^ T_F_ cells, and CD25^−^ LAG3^−^ T_FH_ cells (Fig. 4 a), and performed RNA sequencing (RNA-seq). Paired analyses from three different tonsil donors revealed that CD25^+^ T_F_ cells were fundamentally different from naive, T_FH_, and LAG3^+^ T_FH_ cells (Fig. 4 b) and were remarkably similar to the phenotype described for mouse T_FR_ cells (FOXP3^+^ T_F_ cells). Indeed, human CD25^+^ T_F_ cells expressed key molecules required for T_FH_ development, including *BCL6*, and showed the highest expression of transcripts associated with effector T reg cells including *CTLA4*, glucocorticoid-induced TNFR-related protein (*GITR*), PR domain zinc finger protein 1 (*PRDM1*), Runt-related transcription factor 2 (*RUNX2*), C-C chemokine receptor type 5 (*CCR5*), and *IL-10* (Fig. 4 c; Fu et al., 2012). Similar to mouse T_FR_ cells, human CD25^+^ T_F_ cells expressed the lowest amount of the key B cell helper molecule *CD40L* but abundant IL-21 transcript and protein (Fig. S2, a and b), which is low in mouse T_FR_ cells but has also been shown to be expressed in T_FR_ cells from macaques (Chowdhury et al., 2015).

Flow cytometric analysis of protein expression validated our transcriptomic analyses and confirmed the similarities between CD25^+^ T_F_ cells and T reg cells (Fig. 4 d). Also, minimal differences were observed between the small fraction of tonsil follicular FOXP3^+^ CD25^+^ and the more abundant FOXP3^−^ CD25^+^ T_F_ cells. The latter expressed more IL-10 and BCL6, whereas FOXP3^+^ cells expressed more HELIOS and GITR (both direct targets of FOXP3; Fig. S3). Together, these results suggest that human CD25^+^ T_F_ cells have a gene expression profile that allows migration to facilitate T:B cell encounters and T reg cell function as well as abundant expression of IL-10.

### Human CD25^+^ T_F_ cells regulate T_FH_ cells and B cell IgE production

**Figure 5.**
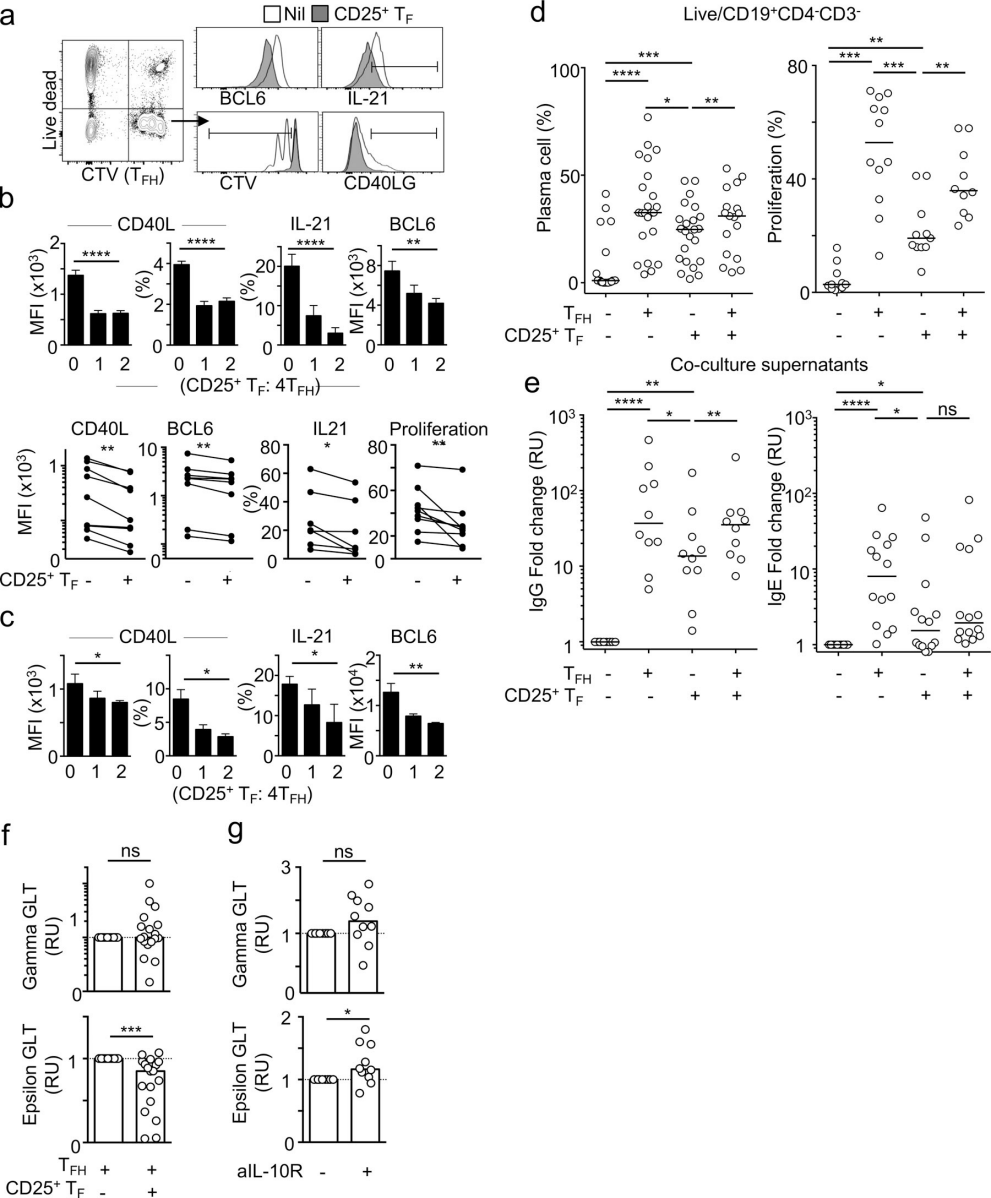
**Human CD25^+^ T_F_ cells repress T_FH_ cells and induction of IgE switching. (a and b)** Flow cytometric plots (a) and quantification (*n* = 8; b) of CTV-labeled-T_FH_ cells, cocultured with memory B cells with or without CD25^+^ T_F_ cells, showing expression of the indicated proteins after 3 d. Upper panels are representative data for a single donor, and lower panels are pooled values from different donors and experiments. Data in each panel is representative of at least five independent experiments. **(c)** Flow cytometric plots and quantification of CTV-labeled T_FH_ cells, cocultured with or without CD25^+^ T_F_ cells, showing expression of the indicated proteins after 3 d of α-CD3 and α-CD28 stimulation. **(d)** Quantification of plasma cell (CD27^+^ CD38^+^) differentiation (*n* = 25) or proliferation (*n* = 12) from memory B cells cocultured with T_FH_ cells, CD25^+^ T_F_ cells, or both in the presence of SEB (500 ng/ml), IL-4, and IL-13 (40 ng/ml) for 7 d. **(e)** IgG (*n* = 10) or IgE (*n* = 14) in coculture supernatants of cocultures as in panel d. Data were normalized to values from cultures without T cells. Data in d and e were pooled from eight independent experiments. **(f and g)** Quantification of γGLTs (top) or εGLTs via qPCR in naive B cells incubated with T_FH_ cells with or without CD25^+^ T_F_ cells (*n* = 19; f), and naive B cells cocultured with CD25^+^ T_F_ cells in the presence or absence of an IL-10 blocking antibody (*n* = 10; g). GLT expression values were calculated using the ΔΔCT method and were normalized to *RPL13A* levels, then normalized to the untreated control. Data were pooled from five independent experiments. Bars represent means of three technical replicates (b and c) or medians (d–g), each dot represents a single tonsil donor, and error bars represent standard deviations. ns, not significant; *, P ≤ 0.05; **, P ≤ 0.01; ***, P ≤ 0.001; ****, P ≤ 0.0001, two-tailed Student’s *t* test (b, top panels, and c–g) and nonparametric paired Wilcoxon test (b, bottom panels). All data are representative of at least three independent experiments. MFI, mean fluorescence intensity; RU, relative units.

In mice, both B and T_FH_ cells have been suggested to be targets of T_FR_ cell suppression (Sage and Sharpe, 2016). To test whether CD25^+^ T_F_ cells could regulate T_FH_ cell function, we analyzed T_FH_ cells after 3 d in culture with autologous memory B cells and CD25^+^ T_F_ cells in the presence of *Staphylococcus* enterotoxin B (SEB). Addition of CD25^+^ T_F_ cells suppressed T_FH_ cell proliferation (Fig. 5 a). In addition, we observed that CD25^+^ T_F_:T_FH_ cocultures reduced the percentage of CD40L-expressing T_FH_ cells (P < 0.0001) and also reduced CD40L expression per T_FH_ cell by 50% (P < 0.0001; Fig. 5 b), as well as IL-21 production (P < 0.0001) and BCL6 expression (P = 0.0010; Fig. 5 b). Reduction of each of these T_FH_ cell molecules is known to limit T_FH_ cell help for B cells (Vinuesa et al., 2016). Similar results were obtained in cultures in which B cells were not included and T_FH_ cells were activated with αCD3/αCD28 antibodies (Fig. 5 c), suggesting that CD25^+^ T_F_ cells act directly on T_FH_ cells to suppress their function. These regulatory effects were not IL-10 mediated, as addition of an IL-10 blocking antibody did not rescue CD25^+^ T_F_ cell–mediated repression of T_FH_ cell proliferation or expression of CD40L, IL-21, or BCL6 (Fig. S4 a).

Next, we compared the ability of human T_FH_ and CD25^+^ T_F_ cells to induce B cell responses. Human memory B cells were cocultured with autologous T_FH_ or CD25^+^ T_F_ cells for 7 d in the presence of SEB. Coculture of B cells with T_FH_ cells successfully induced cell division, differentiation of plasma cells (Fig. 5 d), and secretion of IgG and IgE (Fig. 5 e). Coculture with CD25^+^ T_F_ cells induced plasma cell differentiation, although to a lesser extent (Fig. 5 d, left), and modest B cell proliferation (Fig. 5 d, right) and resulted in a 5.2-fold decrease in IgE secretion and a 2.7-fold decrease in IgG compared with T_FH_ cocultures (Fig. 5 e). Addition of T_FH_ cells to the CD25^+^ T_F_:B cell cocultures at equal ratios rescued plasma cell production, B cell proliferation, and IgG production, but not IgE production (Fig. 5, d and e). Together, these data suggest that human CD25^+^ T_F_ cells are able to regulate T_FH_ cell function and B cell IgE production.

### Human CD25^+^ T_F_ cell–derived IL-10 represses epsilon germline transcription

Early reports from human in vitro studies showed that IL-10 could suppress class-switching to IgE but not IgG (Jeannin et al., 1998; Akdis and Blaser, 2001). Since IL-10 can have additional effects on B cells such as promoting plasma cell differentiation (Arpin et al., 1995), we sought to separate the observed action of CD25^+^ T_F_ cells on plasma cell induction (Fig. 5 d) from possible repressive effects on Ig switching to IgE by looking at the earliest event that occurs in cells undergoing CSR: production of germline transcripts (GLTs). To investigate direct CSR events to IgE from IgM^+^ B cells as opposed to sequential CSR, in which an initial IgM-to-IgG1 CSR event is followed by switching to IgE, we limited our culture to 25 h. This is sufficient to induce the production of εGLTs, but is not sufficient for naive B cells to undergo two cell divisions, thus preventing sequential CSR from occurring (Erazo et al., 2007). Naive CD19^+^ IgD^+^ human B cells were purified and cocultured with T_FH_ cells and stimulated with IL-4, IL-13, and SEB in the presence or absence of CD25^+^ T_F_ cells. Although addition of CD25^+^ T_F_ cells did not have a statistically significant effect on γGLT induction, it consistently reduced εGLTs in T_FH_:B cell cocultures (Fig. 5 f). While there was high variability across individuals, paired statistics revealed a reduction in εGLT across 18 human donors (P = 0.0006). CD25^+^ T_F_ cells repressed εGLTs in IgM^+^ but not in IgG^+^ memory B cells (Fig. S4 b), suggesting that CD25^+^ T_F_ cells preferentially repress direct as opposed to sequential induction of CSR to IgE, or that CD25^+^ T_F_ cells preferentially interact with IgM^+^ B cells (naive or memory).

IL-10 has been shown to suppress εGLTs in human B cell cultures (Jeannin et al., 1998). We therefore evaluated the contribution of IL-10 to CD25^+^ T_F_ cell–mediated suppression of εGLTs. Blocking the IL-10 receptor (Fig. S4 c) did not have a profound effect on γGLT but rescued εGLTs in CD25^+^ T_F_:B cell cocultures (Fig. 5 g) in 8 out of 10 donors. Taken together, these data suggest that IL-10 contributes to suppression of IgE production by human CD25^+^ T_F_ cells.

### IL-10–producing CD25^+^ T_F_ cells inversely correlate with circulating IgE in the serum

**Figure 6.**
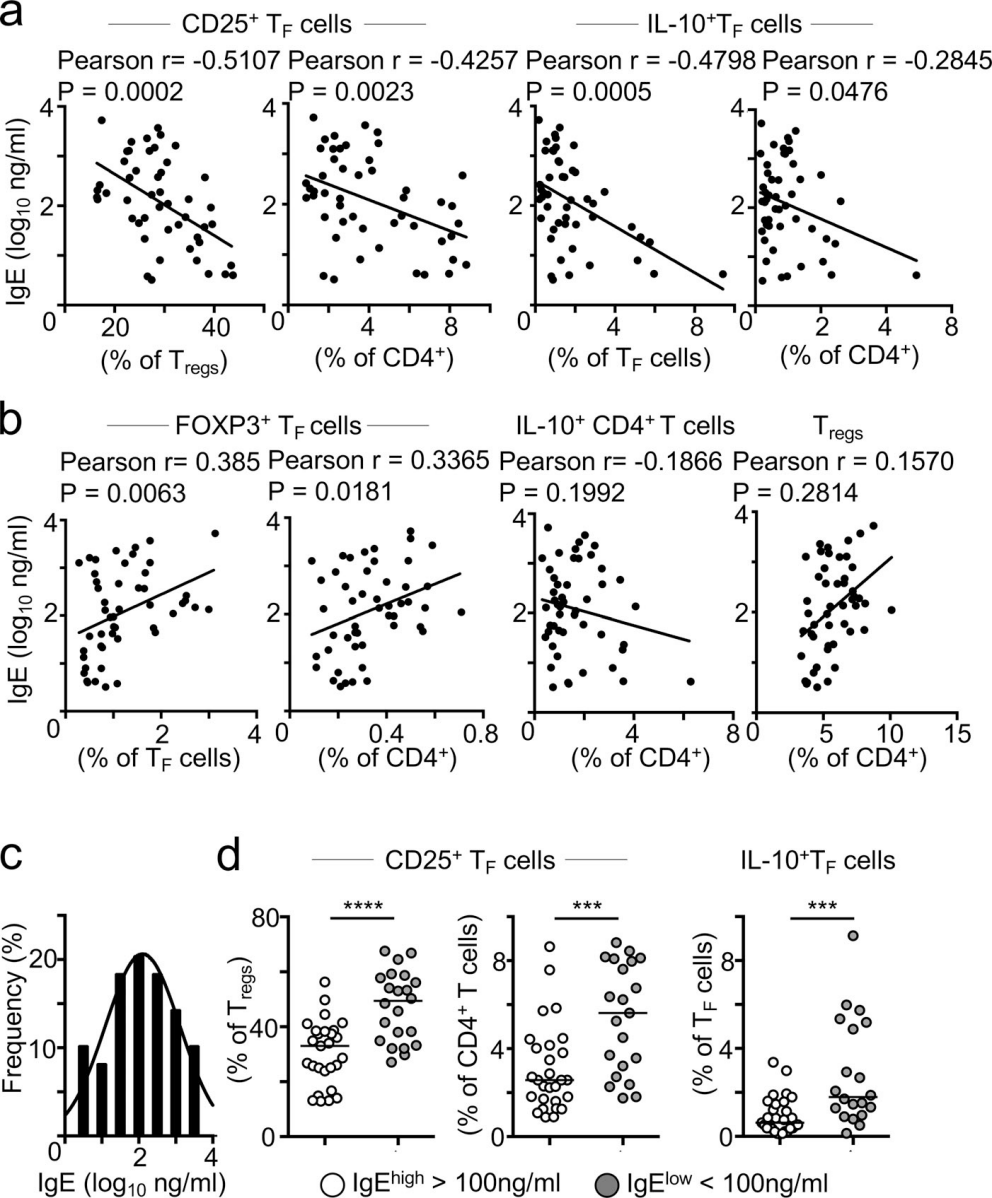
**Tonsillar CD25^+^ T_F_ cells inversely correlate to total IgE in the serum. (a and b)** Pearson correlation analyses between serum total IgE and the frequency of the indicated cell subset in tonsil (*n* = 49). Data are representative of two independent experiments that were pooled together. **(c)** Histogram showing the frequency distribution of serum total IgE (log_10_ ng/ml) from 49 children (mean = 2.07). **(d)** Quantification of the frequency of the indicated cell subset from tonsil donors with high (>100 ng/ml) or low (<100 ng/ml) total serum IgE titers. Bars represent medians, and dots represent individual tonsil donors (*n* = 49). Data are representative of two independent experiments that were pooled together. ***, P ≤ 0.001; ****, P ≤ 0.0001, nonparametric Mann–Whitney test *U* test.

To gain some insights into the possible clinical relevance of CD25^+^ T_F_ cells, we collected blood from pediatric tonsil donors at the time of tonsillectomy and produced a cohort of matched serum and tonsil samples from 50 individuals. We then investigated associations between total IgE in serum and CD25^+^ T_F_ cells in tonsils. Strikingly, we observed an inverse correlation between the frequency of CD25^+^ T_F_ cells, measured as either a percentage of T reg cells or of CD4^+^ T cells, and the amount of IgE in serum (Fig. 6 a). The more frequent CD25^+^ T_F_ cells were, the less abundant total IgE in serum, suggesting that CD25^+^ T_F_ cells may indeed regulate IgE production. Although the goodness of fit in this model was somewhat weak (*r* = 0.5107), the deviation from zero in the correlation was highly significant, demonstrating a negative correlation between the two variables. Similar results were obtained when we correlated the amount of total IgE and the frequency of IL-10–producing T_F_ cells (Fig. 6 a). Compared with CD25^+^ T_F_ cells that lack FOXP3 expression, no negative correlation was observed between serum IgE and the frequency of FOXP3^+^ T_FR_ cells; in fact, a positive correlation was seen (Fig. 6 b, left). No correlations were detected between serum IgE and total T reg cells or even total IL-10–producing CD4^+^ T cells (Fig. 6 b, right), suggesting that CD25^+^ T_F_ cells are the cells specialized in preventing unwanted IgE production.

Given that IL-10 has been reported to cooperate with IL-4 in inducing B cell production of IgG4, and that the latter correlates with allergy desensitization and tolerance, we asked whether CD25^+^ T_F_ cells could also regulate IgG4 responses (Akdis and Akdis, 2014). Pearson correlation analysis revealed no relationship between circulating IgG4 and the frequency of CD25^+^ T_F_ cells or IL-10–producing T_F_ cells (Fig. S5 a). A positive correlation was observed between the frequency of T reg cells and IgG4 (Fig. S5 b), possibly suggesting a division of labor between T reg cells and CD25^+^ T_F_ cells in promoting tolerogenic IgG4 versus repressing IgE responses.

Finally, we asked whether the frequency of CD25^+^ T_F_ cells differed between those donors who exhibited high amounts of IgE versus those with low titers. The clinical value for normal total IgE in children ranges from 2 to 200 IU, or 2.4 to 480 ng/ml (Martins et al., 2014). An average of total serum IgE in healthy individuals of 100 ng/ml has been reported (Gould, 1998) and was the average value found in our tonsil/serum cohort (Fig. 6 c). We selected 100 ng/ml as a cutoff to separate tonsil donors with high versus low IgE titers and thus interrogated whether the frequency of CD25^+^ T_F_ cells differed in both groups. CD25^+^ T_F_ cells, as a percentage of T reg cells or CD4 T cells, were significantly less frequent in donors with high IgE titers (62% and 42% median decrease, respectively; Fig. 6 d). Similarly, the frequency of IL-10–producing T_F_ cells was more abundant in donors with low IgE titers. Altogether, these results show an inverse correlation between CD25^+^ T_F_ cells and serum IgE, suggesting that CD25^+^ T_F_ cells are physiological regulators of IgE production.

## Discussion

Here we have described a novel regulatory function of a unique human T_F_ cell subset that is abundant in tonsil, CD25^+^ T_F_ cells, and is the major IL-10–producing subset among T_F_ cells. CD25^+^ T_F_ cells are identified by high expression of the GC T_FH_ cell markers PD-1 and CXCR5 together with T reg cell markers (CD25^hi^ CD127^lo^ CTLA4^hi^ IL-2^lo^) and high IL-10 production in the absence of FOXP3 expression. Several lines of evidence indicate that this subset is functionally related to FOXP3-expressing T reg cells. First, our transcriptomic and proteomic characterization of CD25^+^ T_F_ cells has revealed that they closely resemble mouse T_FR_ cells, except for the absence of FOXP3 expression and IL-21 production. A CD25^+^ nonfollicular T reg cell population that also lacks FOXP3 expression and produces IL-10 has been previously described (Facciotti et al., 2016). These cells regulate B cell responses and, when dysregulated, are associated with autoimmune diseases. Second, besides high CTLA4 expression, CD25^+^ T_F_ cells express low levels of IL-2. These observations are consistent with previous evidence that T cells engineered to express CTLA4 and not IL-2 adopt full T reg cell–like activity even in the absence of FOXP3 (Yamaguchi et al., 2013). Absence of demethylation in the *FOXP*3 CNS2 promoter, together with low HELIOS expression, suggests that this subset may be induced in the periphery, arising from conventional naive CD4^+^ T cells or from T_FH_ cells themselves.

Functionally, we show that human CD25^+^ T_F_ cells exert suppressive effects on both T and B cells. With respect to T cells, CD25^+^ T_F_ cells repress total T cell and T_FH_ cell proliferation and function. CD25^+^ T_F_ cells dampen T_FH_ cell help through direct down-regulation of CD40L, IL-21, and BCL6 on T_FH_ cells. CD25^+^ T_F_ cells also exert regulatory effects on B cells. First, CD25^+^ T_F_ cells appear to dampen B cell CSR to IgE. The latter occurs in an IL-10–dependent manner. Indeed, CD25^+^ T_F_ cells reduce εGLT production in T_FH_:B cell cocultures, and this effect is rescued by IL-10 blockade. This is interesting, because IL-10 acts very early, within 25 h of naive (unswitched) B cell activation, to suppress CSR by inhibiting εGLT production. Our experimental system shows this effect is a direct consequence of limiting direct CSR from μ to ε, rather than inhibiting γGLT production and subsequent sequential class-switching. It is still unclear whether CD25^+^ T_F_ cells predominantly target naive, GC B, or memory B cells in vivo. T_FH_ cells influence B cell activation at multiple stages, from the earliest B cell priming stages at the T:B border where Ig CSR is initially triggered (Jacob et al., 1991; Toellner et al., 1996), to selection in GCs, and reactivation as memory B cells (Vinuesa et al., 2016). Thus, CD25^+^ T_F_ cells are also likely to interact with and influence naive and memory B cell isotype switching.

Interestingly, and as shown for mouse IL-10–producing T_F_ cells, CD25^+^ T_F_ cells induced some plasma cell differentiation, although to a lesser extent than T_FH_ cells. This contrasts with mouse T_FR_ cells that effectively repress B cell differentiation (Sage et al., 2016; Botta et al., 2017; Maceiras et al., 2017). One obvious difference between mouse (FOXP3^+^) T_FR_ cells and the human tonsillar FOXP3^−^ CD25^+^ T_F_ cells described here is the production of IL-21 by the latter but not the former. IL-21 is known to promote plasma cell differentiation in mice (Ozaki et al., 2002) and humans (Ettinger et al., 2005), so it is possible that this cytokine contributes to plasma cell differentiation, potentially in conjunction with CD25^+^ T_F_ cell–derived IL-10. Although we could not see effects of IL-10R blockade on plasma cell differentiation in 7-d cultures (unpublished data), it is possible that the conditions were not optimal for durable IL-10 blockade in these assays. Interestingly, unlike in mice, in which IL-21 inhibits IgE formation (Ozaki et al., 2002), IL-21 is a potent inducer of IgE in CD40L-stimulated human B cells (Berglund et al., 2013). Thus, there appears to be dissociation between the effects of CD25^+^ T_F_ cells on plasma cell formation, possibly conferred by IL-21 with or without contribution of IL-10, and CSR conferred by IL-10. CD25^+^ T_F_ cells emerge as a unique regulatory cell that controls switching independently of B cell differentiation.

Thymus-derived T reg cells that stably express FOXP3 are selected on the basis of their ability to recognize self-antigen in the thymus (Hsieh et al., 2012). T reg cells give rise to T_FR_ cells in immunized mice (Chung et al., 2011; Linterman et al., 2011; Wollenberg et al., 2011; Sage et al., 2014; Wing et al., 2014; Aloulou et al., 2016), making them effective suppressors of responses against self. Recent reports have also suggested mouse T_FR_ cells can be induced in the periphery in responses to both self and foreign antigens (Aloulou et al., 2016). The human CD25^+^ T_F_ cells described in this study lack FOXP3 expression and appear not to be thymus derived. The fact that CD25^+^ T_F_ cells are so abundant in tonsils, which are exposed to oral and inhaled antigens, but are scarce in other lymphoid tissues that are not exposed to such antigens, makes them particularly good candidates to suppress responses to harmless foreign antigens. Furthermore, our observation that CD25^+^ T_F_ cells repress CSR to IgE and are associated with lower circulating IgE titers in children undergoing tonsillectomy suggests that this subset may be important to prevent IgE-mediated allergic reactions to inhaled or ingested antigens.

Besides a role in preventing immune responses and proallergenic IgE to innocuous antigens, the chronic and ongoing nature of GC reactions in human tonsils makes this subset highly suitable to control excessive and harmful inflammation to foreign antigens, thus serving as a natural homeostatic mechanism to curtail T_FH_ cell responses. Equivalent T reg cell subsets have been described that are specialized in repressing T_H_2 and T_H_1 lineages (Jankovic et al., 2007; Altin et al., 2012; Josefowicz et al., 2012b). There is evidence that T_FR_ cells can be induced against both self and foreign antigens (Aloulou et al., 2016), but it remains unclear whether self- or innocuous environmental antigens such as allergens are more conducive to T_FR_ or CD25^+^ T_F_ cell development.

In summary, our work unveils a novel subset of T_FR_ cells in humans, characterized by abundant production of IL-10, which regulates several aspects of T and B cell responses and prevents induction of switching to IgE. Importantly, low numbers of CD25^+^ T_F_ cells are associated with elevated circulating IgE levels. We therefore predict that deficiencies in homeostasis or function of this cell subset could underpin susceptibility to allergic and anaphylactic reactions induced by inhaled and ingested antigens. Should this be the case, these findings may open up new avenues for boosting CD25^+^ T_F_ cells to reduce the risk of allergy.

## Materials and methods

### Human tonsil and lymph node cells

Human tonsils were obtained from children undergoing routine tonsillectomy. Tonsillar lymphocyte single-cell suspensions were prepared by mechanical disruption of the tissue followed by cell separation using Ficoll-Hypaque (GE Healthcare Life Sciences) gradient and frozen until further use, except for RNA-seq, for which fresh samples were used. Human mesenteric lymph nodes were obtained as discarded tissue from nonmalignancy gastrointestinal surgery. Informed consent was obtained from all subjects. All experiments with human samples were approved by the Australian National University's Human Experimentation Ethics Committee and the Australian Capital Territory Health Human Research Ethics Committee.

### Flow cytometry

Tonsillar lymphocytes were stained with the following anti-human antibodies: anti-CADM1 biotin (3E1; MBL), anti-CD4 APCCy7 (RPA-T4; BD Biosciences), anti-CD8 FITC (RPA-T8; BD Biosciences) or PE (SK1; BD Biosciences), anti-CD19 FITC (SJ25C1; BioLegend) or PE Cy7 (SJ25C1; BD Biosciences), anti-CXCR5 Alexa Fluor 488 or 647 or PerCPCy5.5 (RF8B2; BD Biosciences) or PerCP/Cy5.5 (J252D4; BioLegend), anti-CD45 RA (HI100; BioLegend), anti-CD45RO (UCHL1; BioLegend), anti–CTLA-4 PE (BNI3; BD Biosciences) or PE Cy7 (L3D10; BioLegend), anti–PD-1 PE (MIH4; eBioscience) or BV605 or BV421 (EH12.2H7; BioLegend) or unconjugated (J105; eBioscience), anti-CD127 FITC (11-1278; eBioscience) or BV421 (A019D5; BioLegend), anti-CD25 biotin (BC96; eBioscience or BioLegend) or PE-Cy7 (BC96; BD Biosciences or BioLegend) or APC (2A3; BD Biosciences), anti-CD127 FITC (A019D5; eBioscience) or BV421 (A109D5; BioLegend) or BV510 (HIL-7R-M21; BD Biosciences), anti-FOXP3 Alexa Fluor 647 (259D; eBioscience), anti-GITR PE (110416; R&D Systems), anti–CTLA-4 PE-CF-594 (BNI3; BD Biosciences) or PE Cy7 (L3D10; BioLegend), anti-CD40L Pacific Blue or FITC (24-31; BioLegend), anti-BCL6 Alexa Fluor 647 or PE-Cy7 (K112-91; BD Biosciences), anti–BLIMP-1 Alexa Fluor 647 (646702; R&D Systems), anti-CXCL13 APC (53610; R&D Systems), anti-Helios PE (22F6; BioLegend), anti–IL-10 PE (JES3-19F1; BioLegend) or APC (JES3-19F1; BD Biosciences), anti–IL-10R PE (3F9; iCyt), anti–IL-21 PE (3A3-N21; BD Biosciences), anti-IL2 Alexa Fluor 488 (MQ1-17H12; BioLegend), anti-CD3 APC (HIT3a; BD Biosciences) or Alexa Fluor 700 (UCHT1; BD Biosciences) or Pacific Blue (OKT3; BioLegend) or BV510 (OKT3; BioLegend), anti-CD27 FITC or APC (M-T271; BD Biosciences), anti-CD38 FITC (HIT2; BD Biosciences) or PE (HB7; BD Biosciences), anti-LAG3 PE or FITC (2319-L3-050; R&D Systems), and anti-pSTAT3 Alexa Fluor 647 (4/P-STAT3; BD Biosciences). Intracellular staining was performed using the FOXP3/Transcription Factor Staining Buffer Set (eBioscience) or Cytofix/Cytoperm (BD Biosciences) according to the manufacturer’s instructions. LAG3 was stained at 37°C for 15 min in the dark.

Mesenteric lymph nodes were stained with the following anti-human antibodies: anti-CD3 BUV395 (UCHT1; BD Biosciences), anti-CD4 PerCP (RPA-T4; BioLegend), anti-CD8 APC-C7 (RPA-T8; BioLegend), anti-CD19 APC-Cy7 (HIB19; BioLegend), anti-CD45RA PE-TR (MEM-56; Invitrogen), anti-CXCR5 BV510 (RF8B2; BD Biosciences), anti–PD-1 BV421 (EH12.2H7; BioLegend), anti-CD127 BV650 (HIL-7R-M21; BD Biosciences), anti-CD25 PE-C7 (BC96; BioLegend), anti-FOXP3 PE (259D/C7; BD Biosciences), and anti-CD27 BV711 (L128; BD Biosciences). Intracellular staining was performed using the FOXP3/Transcription Factor Staining Buffer Set according to the manufacturer’s instructions.

### CpG methylation analysis by bisulfite sequencing

Genomic DNA was prepared using the NucleoSpin Tissue XS kit (Macherey-Nagel). After sodium bisulfite treatment (MethylEasy Xceed, Human Genetic Signatures), modified DNA was amplified by PCR and subcloned into PCR2.1-TOPO Vector (Invitrogen). PCR primers used were 5′-TTG​GGT​TAA​GTT​TGT​TGT​AGG​ATA​G-3′ and 5′-ATC​TAA​ACC​CTA​TTA​TCA​CAA​CCC​C-3′. The colonies (16–48 colonies/region) were directly amplified using the Illustra TempliPhi Amplification Kit (GE Healthcare) and sequenced.

### In vitro stimulation

Intracellular cytokine staining was performed following 4–6 h of PMA (50 ng/ml) and ionomycin (500 ng/ml) stimulation with GolgiStop (BD Biosciences) or Brefeldin A (BioLegend) in RPMI 1640 medium supplemented with 10% FCS, 2 mM l-glutamine, 100 U penicillin-streptomycin, 0.1 mM nonessential amino acids, 100 mM Hepes, and 44 µM 2-mercaptoethanol. 200,000 naive B cells were FACS-purified and stimulated with 50 ng/ml recombinant human IL-10 (Peprotech) for 30 min with or without 5 µg/ml of anti–IL-10 blocking antibody (3F9; BioLegend).

### Secreted cytokine surface capture

Live CD4^+^ human tonsillar lymphocytes were stained with the anti-human IL-10 catch reagent (Miltenyi) at 9 million cells per 100 µl. After 10 min on ice, the cells were diluted 1:20 in RPMI supplemented as above. Cells were stimulated with PMA (100 ng/ml) and ionomycin (500 ng/ml) and incubated for 2 h in a 37°C incubator with 5% CO_2_, rotating slowly using a MacsMix (Miltenyi). Cells were then stained as above using the anti-human IL-10 detection antibody (Miltenyi).

### Microarray RNA analysis

IL-10–positive and –negative CD25^+^ T_F_ cells were FACS-purified from three human subjects. mRNA was extracted, and samples were sent to the Ramaciotti Centre for Genomics (Sydney, Australia) for analysis with Affymetrix GeneChip Human Gene 2.0 ST microarrays. The resulting nine CEL files were imported into Partek Genomics Suite (version 6.6) using the RMA algorithm with RMA background correction, quantile normalization, and median polish probe set summarization. Differentially expressed probe sets were identified in Partek GS using a two-factor ANOVA model with the factor SubjectID. The data are deposited under the National Center for Biotechnology Information Gene Expression Omnibus accession no. GSE79887.

### Autologous human tonsillar T_FH_:B cell cocultures and IgE detection

20,000 human FACS-purified T_FH_ and/or CD25^+^ T_F_ cells were cocultured with 100,000 GC B (CD4^−^ CD19^+^ CD38^+^ CD27^int^) cells or memory B (CD4^−^ CD19^+^ CD38^−^ CD27^+^) cells in the presence of SEB (500 ng/ml; Sigma-Aldrich) for 3, 5, or 7 d. For IgE-inducing conditions, recombinant IL-4 and IL-13 (Peprotech) were used at 40 ng/ml. Culture supernatants, or human serum, were used to measure IgE, IgG, and IgG4 using the Cytometric Bead Array flex sets (558682, 558679, and 558678; BD Bioscience) according to the manufacturer’s instructions. For proliferation assays, cells were stained with CellTrace Violet (CTV; Thermo Fisher Scientific) according to manufacturer’s instructions. IL-10R blockade was achieved using anti–IL-10R blocking antibody (3F9; BioLegend) or isotype control (RTK2758; BioLegend) at 5 µg/ml. For εGLT detection, 100,000 naive B cells (IgD^+^ CD19^+^ cells) were incubated with recombinant IL-4 and IL-13 together with SEB with or without T_FH_ or CD25^+^ T_F_ cells for 24–25 h. RNA was extracted using phenol/chloroform extraction, and cDNA was synthesized using SuperScript III (Invitrogen), according to the manufacturer’s instructions. The following trancripts were measured via qPCR and analyzed using the ΔΔCT method. εGLTs were detected using the following primers (forward: 5′-TGC​ATC​CAC​AGG​CAC​CAA​AT-3′, reverse: 5′-ATC​ACC​GGC​TCC​GGG​AAG​TA-3) and normalized to *RPL13A* expression levels (forward: 5′-CTC​AAG​GTG​TTT​GAC​GGC​ATC​C-3′, reverse: 5′-TAC​TTC​CAG​CCA​ACC​TCG​TGA​G-3′). γGLTs were measured by using the following primers (forward: 5′-TCC​AAG​CCA​ACA​GGG​CAG​GAC​ACA​CCC​AGA​G-3′, reverse: 5′-AAG​TAG​TCC​TTG​ACC​AGG​CAG-3′).

### Human T reg cell suppression assays

10,000 CTV-labeled FACS-purified responder T cells (CD4^+^ CD3^+^ CD25^−^) were cocultured in the presence or absence of serially diluted follicular or nonfollicular T reg cells starting with 20,000 cells. Cells were stimulated with αCD3/αCD28 microbeads (Miltenyi) at a 1:1 ratio of beads to T responder cells. After 3 d, cells were stained with 7-aminoactinomycin D and then analyzed by flow cytometry.

### RNA-seq and analysis

The different subsets of human T_F_ cells were FACS-purified from three fresh tonsils. mRNA was extracted and sent to the Australian Cancer Research Foundation Biomolecular Resource Facility, The John Curtin School of Medical Research, The Australian National University (ANU; Canberra, Australia), for library construction using the TruSeq Stranded mRNA LT Sample Prep Kit (Illumina). Library samples were sequenced on a HiSeq2000 machine with a coverage of 25 million reads. The data were then sent to the Genome Discovery Unit (ANU Bioinformatics Consulting Unit) for analysis. Initial quality control checks with FastQC revealed that none of the 12 samples were problematic. All reads were aligned to the *Homo sapiens* genome reference sequence using TopHat version 2.0.13 with default parameters. Read counts were then generated for each gene in each sample using featureCounts version 1.4.6-p1 by using annotated gene locations. Differential expression analysis was performed using the edgeR package, version 3.10. Read counts per gene were normalized by trimmed mean of M-values. Because edgeR uses the negative binomial distribution as its basic model for differential expression data, dispersion estimates were obtained using the quantile-adjusted conditional maximum likelihood method for single-factor experiments. Then, the quantile-adjusted conditional maximum likelihood–based exact test for the negative binomial distribution was performed to test for differentially expressed genes in our groups of samples. We used a Benjamini–Hochberg adjusted P value threshold of 0.05 to identify significantly differentially regulated genes. Data are deposited under National Center for Biotechnology Information Sequence Read Archive accession no. SRP072739.

### Statistical analyses

All data were analyzed with nonparametric Mann–Whitney *U* test, except for some human cell culture experiments, in which paired Student’s *t* tests or two-way ANOVAs were used. Paired analyses were performed using the Wilcoxon test. All statistical analyses were performed with Prism software (version 6, GraphPad). Statistically significant differences are indicated as *, P ≤ 0.05; **, P ≤ 0.01; ***, P ≤ 0.001; ****, P ≤ 0.0001; and ns, not significant.

### Online supplemental material

Fig. S1 shows the frequency of CD25^+^ cells in human lymph node tissue. Fig. S2 shows IL-21 production by the different T_F_ cell subsets. Fig. S3 compares T reg cell– and T_FH_ cell–associated protein expression among CD25^+^ T_F_ cells and the more classical FOXP3^+^ T_FR_ cells. Fig. S4 shows that CD25^+^ T_F_-mediated repression of T_FH_ cells is IL-10 independent, as well as repression of εGLTs in IgG^+^ and IgM^+^ memory B cells. Fig. S5 shows Pearson correlation analyses between the frequency of CD25^+^ T_F_ cells in tonsil versus IgG4 serum titers.

## Supplementary Material

Supplemental Materials (PDF)

## References

[bib1] Akdis C.A., and Akdis M. 2014 Mechanisms of immune tolerance to allergens: role of IL-10 and Tregs. J. Clin. Invest. 124:4678–4680. 10.1172/JCI78891 25365074 PMC4347251

[bib2] Akdis C.A., and Blaser K. 2001 Mechanisms of interleukin-10-mediated immune suppression. Immunology. 103:131–136. 10.1046/j.1365-2567.2001.01235.x 11412299 PMC1783236

[bib3] Aloulou M., Carr E.J., Gador M., Bignon A., Liblau R.S., Fazilleau N., and Linterman M.A. 2016 Follicular regulatory T cells can be specific for the immunizing antigen and derive from naive T cells. Nat. Commun. 7:10579 10.1038/ncomms10579 26818004 PMC4738360

[bib4] Altin J.A., Goodnow C.C., and Cook M.C. 2012 IL-10+ CTLA-4+ Th2 inhibitory cells form in a Foxp3-independent, IL-2-dependent manner from Th2 effectors during chronic inflammation. J. Immunol. 188:5478–5488. 10.4049/jimmunol.1102994 22547705

[bib5] Arpin C., Déchanet J., Van Kooten C., Merville P., Grouard G., Brière F., Banchereau J., and Liu Y.J. 1995 Generation of memory B cells and plasma cells in vitro. Science. 268:720–722. 10.1126/science.7537388 7537388

[bib6] Ballesteros-Tato A., Randall T.D., Lund F.E., Spolski R., Leonard W.J., and León B. 2016 T Follicular Helper Cell Plasticity Shapes Pathogenic T Helper 2 Cell-Mediated Immunity to Inhaled House Dust Mite. Immunity. 44:259–273. 10.1016/j.immuni.2015.11.017 26825674 PMC4758890

[bib7] Berglund L.J., Avery D.T., Ma C.S., Moens L., Deenick E.K., Bustamante J., Boisson-Dupuis S., Wong M., Adelstein S., Arkwright P.D., 2013 IL-21 signalling via STAT3 primes human naive B cells to respond to IL-2 to enhance their differentiation into plasmablasts. Blood. 122:3940–3950. 10.1182/blood-2013-06-506865 24159173 PMC3854113

[bib8] Botta D., Fuller M.J., Marquez-Lago T.T., Bachus H., Bradley J.E., Weinmann A.S., Zajac A.J., Randall T.D., Lund F.E., León B., and Ballesteros-Tato A. 2017 Dynamic regulation of T follicular regulatory cell responses by interleukin 2 during influenza infection. Nat. Immunol. 18:1249–1260. 10.1038/ni.3837 28892471 PMC5679073

[bib9] Butt D., Chan T.D., Bourne K., Hermes J.R., Nguyen A., Statham A., O’Reilly L.A., Strasser A., Price S., Schofield P., 2015 FAS Inactivation Releases Unconventional Germinal Center B Cells that Escape Antigen Control and Drive IgE and Autoantibody Production. Immunity. 42:890–902. 10.1016/j.immuni.2015.04.010 25979420

[bib10] Carreras J., Lopez-Guillermo A., Fox B.C., Colomo L., Martinez A., Roncador G., Montserrat E., Campo E., and Banham A.H. 2006 High numbers of tumor-infiltrating FOXP3-positive regulatory T cells are associated with improved overall survival in follicular lymphoma. Blood. 108:2957–2964. 10.1182/blood-2006-04-018218 16825494

[bib11] Choi Y.S., Gullicksrud J.A., Xing S., Zeng Z., Shan Q., Li F., Love P.E., Peng W., Xue H.H., and Crotty S. 2015 LEF-1 and TCF-1 orchestrate T(FH) differentiation by regulating differentiation circuits upstream of the transcriptional repressor Bcl6. Nat. Immunol. 16:980–990. 10.1038/ni.3226 26214741 PMC4545301

[bib12] Chowdhury A., Del Rio Estrada P.M., Tharp G.K., Trible R.P., Amara R.R., Chahroudi A., Reyes-Teran G., Bosinger S.E., and Silvestri G. 2015 Decreased T Follicular Regulatory Cell/T Follicular Helper Cell (TFH) in Simian Immunodeficiency Virus-Infected Rhesus Macaques May Contribute to Accumulation of TFH in Chronic Infection. J. Immunol. 195:3237–3247. 10.4049/jimmunol.1402701 26297764 PMC4575868

[bib13] Chung Y., Tanaka S., Chu F., Nurieva R.I., Martinez G.J., Rawal S., Wang Y.H., Lim H., Reynolds J.M., Zhou X.H., 2011 Follicular regulatory T cells expressing Foxp3 and Bcl-6 suppress germinal center reactions. Nat. Med. 17:983–988. 10.1038/nm.2426 21785430 PMC3151340

[bib14] Coquet J.M., Schuijs M.J., Smyth M.J., Deswarte K., Beyaert R., Braun H., Boon L., Karlsson Hedestam G.B., Nutt S.L., Hammad H., and Lambrecht B.N. 2015 Interleukin-21-Producing CD4(+) T Cells Promote Type 2 Immunity to House Dust Mites. Immunity. 43:318–330. 10.1016/j.immuni.2015.07.015 26287681

[bib15] Crotty S. 2011 Follicular helper CD4 T cells (TFH). Annu. Rev. Immunol. 29:621–663. 10.1146/annurev-immunol-031210-101400 21314428

[bib16] Erazo A., Kutchukhidze N., Leung M., Christ A.P., Urban J.F. Jr., Curotto de Lafaille M.A., and Lafaille J.J. 2007 Unique maturation program of the IgE response in vivo. Immunity. 26:191–203. 10.1016/j.immuni.2006.12.006 17292640 PMC1892589

[bib17] Ettinger R., Sims G.P., Fairhurst A.-M., Robbins R., da Silva Y.S., Spolski R., Leonard W.J., and Lipsky P.E. 2005 IL-21 induces differentiation of human naive and memory B cells into antibody-secreting plasma cells. J. Immunol. 175:7867–7879. 10.4049/jimmunol.175.12.7867 16339522

[bib18] Facciotti F., Gagliani N., Häringer B., Alfen J.S., Penatti A., Maglie S., Paroni M., Iseppon A., Moro M., Crosti M.C., 2016 IL-10-producing forkhead box protein 3-negative regulatory T cells inhibit B-cell responses and are involved in systemic lupus erythematosus. J. Allergy Clin. Immunol. 137:318–321.e5. 10.1016/j.jaci.2015.06.044 26318071

[bib19] Fonseca V.R., Agua-Doce A., Maceiras A.R., Pierson W., Ribeiro F., Romão V.C., Pires A.R., da Silva S.L., Fonseca J.E., Sousa A.E., 2017 Human blood T_fr_ cells are indicators of ongoing humoral activity not fully licensed with suppressive function. Sci. Immunol. 2:eaan1487 10.1126/sciimmunol.aan1487 28802258 PMC7116402

[bib20] Fu W., Ergun A., Lu T., Hill J.A., Haxhinasto S., Fassett M.S., Gazit R., Adoro S., Glimcher L., Chan S., 2012 A multiply redundant genetic switch ‘locks in’ the transcriptional signature of regulatory T cells. Nat. Immunol. 13:972–980. 10.1038/ni.2420 22961053 PMC3698954

[bib21] Gagliani N., Magnani C.F., Huber S., Gianolini M.E., Pala M., Licona-Limon P., Guo B., Herbert D.R., Bulfone A., Trentini F., 2013 Coexpression of CD49b and LAG-3 identifies human and mouse T regulatory type 1 cells. Nat. Med. 19:739–746. 10.1038/nm.3179 23624599

[bib22] Galli S.J., Tsai M., and Piliponsky A.M. 2008 The development of allergic inflammation. Nature. 454:445–454. 10.1038/nature07204 18650915 PMC3573758

[bib23] Glatman Zaretsky A., Taylor J.J., King I.L., Marshall F.A., Mohrs M., and Pearce E.J. 2009 T follicular helper cells differentiate from Th2 cells in response to helminth antigens. J. Exp. Med. 206:991–999. 10.1084/jem.20090303 19380637 PMC2715032

[bib24] Gould H.J. B.R. 1998 Immunoglobulin E—an overview. *In* Encyclopedia of Immunology, 2nd Ed Academic Press, Cambridge, MA 1202–1208. 10.1006/rwei.1999.0312

[bib25] Hammad H., and Lambrecht B.N. 2015 Barrier Epithelial Cells and the Control of Type 2 Immunity. Immunity. 43:29–40. 10.1016/j.immuni.2015.07.007 26200011

[bib26] He J.-S., Meyer-Hermann M., Xiangying D., Zuan L.Y., Jones L.A., Ramakrishna L., de Vries V.C., Dolpady J., Aina H., Joseph S., 2013 The distinctive germinal center phase of IgE+ B lymphocytes limits their contribution to the classical memory response. J. Exp. Med. 210:2755–2771. 10.1084/jem.20131539 24218137 PMC3832920

[bib27] Hsieh C.S., Lee H.M., and Lio C.W. 2012 Selection of regulatory T cells in the thymus. Nat. Rev. Immunol. 12:157–167. 10.1038/nri3155 22322317

[bib28] Jacob J., Kassir R., and Kelsoe G. 1991 In situ studies of the primary immune response to (4-hydroxy-3-nitrophenyl)acetyl. I. The architecture and dynamics of responding cell populations. J. Exp. Med. 173:1165–1175. 10.1084/jem.173.5.1165 1902502 PMC2118845

[bib29] Jankovic D., Kullberg M.C., Feng C.G., Goldszmid R.S., Collazo C.M., Wilson M., Wynn T.A., Kamanaka M., Flavell R.A., and Sher A. 2007 Conventional T-bet(+)Foxp3(-) Th1 cells are the major source of host-protective regulatory IL-10 during intracellular protozoan infection. J. Exp. Med. 204:273–283. 10.1084/jem.20062175 17283209 PMC2118735

[bib30] Jeannin P., Lecoanet S., Delneste Y., Gauchat J.-F., and Bonnefoy J.-Y. 1998 IgE versus IgG4 production can be differentially regulated by IL-10. J. Immunol. 160:3555–3561. 9531318

[bib31] Josefowicz S.Z., Lu L.F., and Rudensky A.Y. 2012 a Regulatory T cells: mechanisms of differentiation and function. Annu. Rev. Immunol. 30:531–564. 10.1146/annurev.immunol.25.022106.141623 22224781 PMC6066374

[bib32] Josefowicz S.Z., Niec R.E., Kim H.Y., Treuting P., Chinen T., Zheng Y., Umetsu D.T., and Rudensky A.Y. 2012 b Extrathymically generated regulatory T cells control mucosal TH2 inflammation. Nature. 482:395–399. 10.1038/nature10772 22318520 PMC3485072

[bib33] King I.L., and Mohrs M. 2009 IL-4-producing CD4+ T cells in reactive lymph nodes during helminth infection are T follicular helper cells. J. Exp. Med. 206:1001–1007. 10.1084/jem.20090313 19380638 PMC2715031

[bib34] Kobayashi T., Iijima K., Dent A.L., and Kita H. 2017 Follicular helper T cells mediate IgE antibody response to airborne allergens. J. Allergy Clin. Immunol. 139:300–313.e7. 10.1016/j.jaci.2016.04.021 27325434 PMC5115999

[bib35] Komohara Y., Ma C., Yano H., Pan C., Horlad H., Saito Y., Ohnishi K., Fujiwara Y., Okuno Y., Nosaka K., 2017 Cell adhesion molecule-1 (CADM1) expressed on adult T-cell leukemia/lymphoma cells is not involved in the interaction with macrophages. J. Clin. Exp. Hematop. 57:15–20. 10.3960/jslrt.17003 28420814 PMC6144270

[bib36] Lee S.K., Rigby R.J., Zotos D., Tsai L.M., Kawamoto S., Marshall J.L., Ramiscal R.R., Chan T.D., Gatto D., Brink R., 2011 B cell priming for extrafollicular antibody responses requires Bcl-6 expression by T cells. J. Exp. Med. 208:1377–1388. 10.1084/jem.20102065 21708925 PMC3135363

[bib37] Li H., and Pauza C.D. 2015 CD25(+) Bcl6(low) T follicular helper cells provide help to maturing B cells in germinal centers of human tonsil. Eur. J. Immunol. 45:298–308. 10.1002/eji.201444911 25263533 PMC4293275

[bib38] Li Y., Li W., Ying Z., Tian H., Zhu X., Li J., and Li M. 2014 Metastatic heterogeneity of breast cancer cells is associated with expression of a heterogeneous TGFβ-activating miR424-503 gene cluster. Cancer Res. 74:6107–6118. 10.1158/0008-5472.CAN-14-0389 25164015

[bib39] Licona-Limón P., Kim L.K., Palm N.W., and Flavell R.A. 2013 TH2, allergy and group 2 innate lymphoid cells. Nat. Immunol. 14:536–542. 10.1038/ni.2617 23685824

[bib40] Lim H.W., Hillsamer P., and Kim C.H. 2004 Regulatory T cells can migrate to follicles upon T cell activation and suppress GC-Th cells and GC-Th cell-driven B cell responses. J. Clin. Invest. 114:1640–1649. 10.1172/JCI200422325 15578096 PMC529283

[bib41] Linterman M.A., Rigby R.J., Wong R.K., Yu D., Brink R., Cannons J.L., Schwartzberg P.L., Cook M.C., Walters G.D., and Vinuesa C.G. 2009 Follicular helper T cells are required for systemic autoimmunity. J. Exp. Med. 206:561–576. 10.1084/jem.20081886 19221396 PMC2699132

[bib42] Linterman M.A., Pierson W., Lee S.K., Kallies A., Kawamoto S., Rayner T.F., Srivastava M., Divekar D.P., Beaton L., Hogan J.J., 2011 Foxp3+ follicular regulatory T cells control the germinal center response. Nat. Med. 17:975–982. 10.1038/nm.2425 21785433 PMC3182542

[bib43] Maceiras A.R., Almeida S.C.P., Mariotti-Ferrandiz E., Chaara W., Jebbawi F., Six A., Hori S., Klatzmann D., Faro J., and Graca L. 2017 T follicular helper and T follicular regulatory cells have different TCR specificity. Nat. Commun. 8:15067 10.1038/ncomms15067 28429709 PMC5413949

[bib44] Martins T.B., Bandhauer M.E., Bunker A.M., Roberts W.L., and Hill H.R. 2014 New childhood and adult reference intervals for total IgE. J. Allergy Clin. Immunol. 133:589–591. 10.1016/j.jaci.2013.08.037 24139495

[bib45] Ozaki K., Spolski R., Feng C.G., Qi C.F., Cheng J., Sher A., Morse H.C. III, Liu C., Schwartzberg P.L., and Leonard W.J. 2002 A critical role for IL-21 in regulating immunoglobulin production. Science. 298:1630–1634. 10.1126/science.1077002 12446913

[bib46] Reinhardt R.L., Liang H.E., and Locksley R.M. 2009 Cytokine-secreting follicular T cells shape the antibody repertoire. Nat. Immunol. 10:385–393. 10.1038/ni.1715 19252490 PMC2714053

[bib47] Sage P.T., and Sharpe A.H. 2016 T follicular regulatory cells. Immunol. Rev. 271:246–259. 10.1111/imr.12411 27088919

[bib48] Sage P.T., Paterson A.M., Lovitch S.B., and Sharpe A.H. 2014 The coinhibitory receptor CTLA-4 controls B cell responses by modulating T follicular helper, T follicular regulatory, and T regulatory cells. Immunity. 41:1026–1039. 10.1016/j.immuni.2014.12.005 25526313 PMC4309019

[bib49] Sage P.T., Ron-Harel N., Juneja V.R., Sen D.R., Maleri S., Sungnak W., Kuchroo V.K., Haining W.N., Chevrier N., Haigis M., and Sharpe A.H. 2016 Suppression by T_FR_ cells leads to durable and selective inhibition of B cell effector function. Nat. Immunol. 17:1436–1446. 10.1038/ni.3578 27695002 PMC5502675

[bib50] Seddiki N., Santner-Nanan B., Martinson J., Zaunders J., Sasson S., Landay A., Solomon M., Selby W., Alexander S.I., Nanan R., 2006 Expression of interleukin (IL)-2 and IL-7 receptors discriminates between human regulatory and activated T cells. J. Exp. Med. 203:1693–1700. 10.1084/jem.20060468 16818676 PMC2118333

[bib51] Simpson N., Gatenby P.A., Wilson A., Malik S., Fulcher D.A., Tangye S.G., Manku H., Vyse T.J., Roncador G., Huttley G.A., 2010 Expansion of circulating T cells resembling follicular helper T cells is a fixed phenotype that identifies a subset of severe systemic lupus erythematosus. Arthritis Rheum. 62:234–244. 10.1002/art.25032 20039395

[bib52] Szurek E., Cebula A., Wojciech L., Pietrzak M., Rempala G., Kisielow P., and Ignatowicz L. 2015 Differences in Expression Level of Helios and Neuropilin-1 Do Not Distinguish Thymus-Derived from Extrathymically-Induced CD4+Foxp3+ Regulatory T Cells. PLoS One. 10:e0141161 10.1371/journal.pone.0141161 26495986 PMC4619666

[bib53] Thornton A.M., Korty P.E., Tran D.Q., Wohlfert E.A., Murray P.E., Belkaid Y., and Shevach E.M. 2010 Expression of Helios, an Ikaros transcription factor family member, differentiates thymic-derived from peripherally induced Foxp3+ T regulatory cells. J. Immunol. 184:3433–3441. 10.4049/jimmunol.0904028 20181882 PMC3725574

[bib54] Toellner K.M., Gulbranson-Judge A., Taylor D.R., Sze D.M., and MacLennan I.C. 1996 Immunoglobulin switch transcript production in vivo related to the site and time of antigen-specific B cell activation. J. Exp. Med. 183:2303–2312. 10.1084/jem.183.5.2303 8642339 PMC2192570

[bib55] Victora G.D., Schwickert T.A., Fooksman D.R., Kamphorst A.O., Meyer-Hermann M., Dustin M.L., and Nussenzweig M.C. 2010 Germinal center dynamics revealed by multiphoton microscopy with a photoactivatable fluorescent reporter. Cell. 143:592–605. 10.1016/j.cell.2010.10.032 21074050 PMC3035939

[bib56] Vinuesa C.G., Tangye S.G., Moser B., and Mackay C.R. 2005 Follicular B helper T cells in antibody responses and autoimmunity. Nat. Rev. Immunol. 5:853–865. 10.1038/nri1714 16261173

[bib57] Vinuesa C.G., Linterman M.A., Yu D., and MacLennan I.C. 2016 Follicular Helper T Cells. Annu. Rev. Immunol. 34:335–368. 10.1146/annurev-immunol-041015-055605 26907215

[bib58] Voehringer D., Reese T.A., Huang X., Shinkai K., and Locksley R.M. 2006 Type 2 immunity is controlled by IL-4/IL-13 expression in hematopoietic non-eosinophil cells of the innate immune system. J. Exp. Med. 203:1435–1446. 10.1084/jem.20052448 16702603 PMC2118302

[bib59] Wing J.B., Ise W., Kurosaki T., and Sakaguchi S. 2014 Regulatory T cells control antigen-specific expansion of Tfh cell number and humoral immune responses via the coreceptor CTLA-4. Immunity. 41:1013–1025. 10.1016/j.immuni.2014.12.006 25526312

[bib60] Wing J.B., Kitagawa Y., Locci M., Hume H., Tay C., Morita T., Kidani Y., Matsuda K., Inoue T., Kurosaki T., 2017 A distinct subpopulation of CD25^-^ T-follicular regulatory cells localizes in the germinal centers. Proc. Natl. Acad. Sci. USA. 114:E6400–E6409. 10.1073/pnas.1705551114 28698369 PMC5547636

[bib61] Wollenberg I., Agua-Doce A., Hernández A., Almeida C., Oliveira V.G., Faro J., and Graca L. 2011 Regulation of the germinal center reaction by Foxp3+ follicular regulatory T cells. J. Immunol. 187:4553–4560. 10.4049/jimmunol.1101328 21984700

[bib62] Xiong H., Dolpady J., Wabl M., Curotto de Lafaille M.A., and Lafaille J.J. 2012 Sequential class switching is required for the generation of high affinity IgE antibodies. J. Exp. Med. 209:353–364. 10.1084/jem.20111941 22249450 PMC3280879

[bib63] Yamaguchi T., Kishi A., Osaki M., Morikawa H., Prieto-Martin P., Wing K., Saito T., and Sakaguchi S. 2013 Construction of self-recognizing regulatory T cells from conventional T cells by controlling CTLA-4 and IL-2 expression. Proc. Natl. Acad. Sci. USA. 110:E2116–E2125. 10.1073/pnas.1307185110 23690575 PMC3677445

[bib64] Yang Z., Sullivan B.M., and Allen C.D. 2012 Fluorescent in vivo detection reveals that IgE(+) B cells are restrained by an intrinsic cell fate predisposition. Immunity. 36:857–872. 10.1016/j.immuni.2012.02.009 22406270

[bib65] Yang Z., Robinson M.J., Chen X., Smith G.A., Taunton J., Liu W., and Allen C.D.C. 2016 Regulation of B cell fate by chronic activity of the IgE B cell receptor. eLife. 5:e21238 10.7554/eLife.21238 27935477 PMC5207771

[bib66] Yue D., Ciccolini A., Avilla E., and Waserman S. 2018 Food allergy and anaphylaxis. J. Asthma Allergy. 11:111–120. 10.2147/JAA.S162456 29950871 PMC6016602

[bib67] Zheng Y., Josefowicz S., Chaudhry A., Peng X.P., Forbush K., and Rudensky A.Y. 2010 Role of conserved non-coding DNA elements in the Foxp3 gene in regulatory T-cell fate. Nature. 463:808–812. 10.1038/nature08750 20072126 PMC2884187

