# Supplementary material for: Regulatory roles of IL-10–producing human follicular T cells
Source: J Exp Med. 2019 Jun 17;216(8):1843–56. doi: 10.1084/jem.20190493 (PMC6683995; doi:10.1084/jem.20190493)
Supplement: Supplemental Materials (PDF) [file JEM_20190493_sm.pdf]

Supplemental material

Cañete et al., https://doi.org/10.1084/jem.20190493

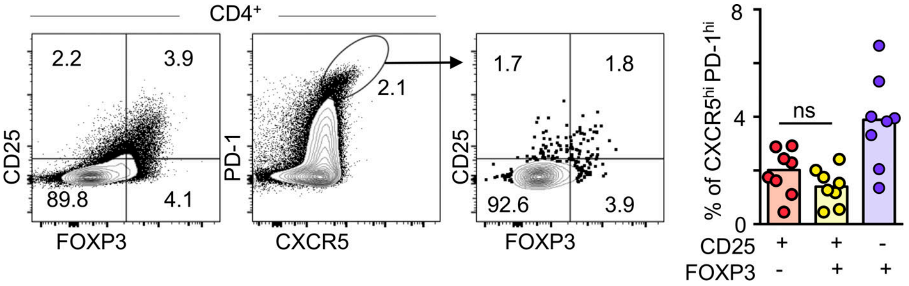

Figure S1. **Human CD25+ T_F cells in human lymph nodes.** Flow cytometric plots and quantification of CD25- and FOXP3-expressing cells among T_F cells from human mesenteric lymph nodes (*n* = 8). Data are representative of two independent experiments. Bars represent medians, and each dot represents a single tonsil donor. ns, not significant, nonparametric Mann–Whitney *U* test.

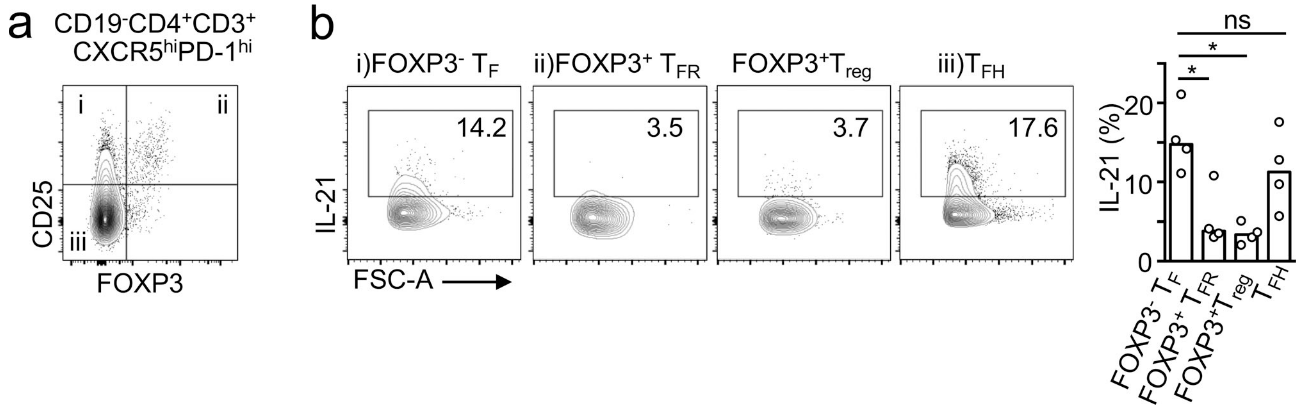

Figure S2. **CD25+ T_F cells express abundant IL-21. (a and b)** Flow cytometric plots and quantification showing gating strategy (a) and IL-21 expression of PMA/ionomycin-stimulated tonsillar cell suspensions in the indicated subset (*n* = 4; b). Data are representative of two independent experiments. Bars represent medians, and each dot represents a single donor. ns, not significant; *, P ≤ 0.05, nonparametric Mann–Whitney *U* test. FSC-A, forward scatter.

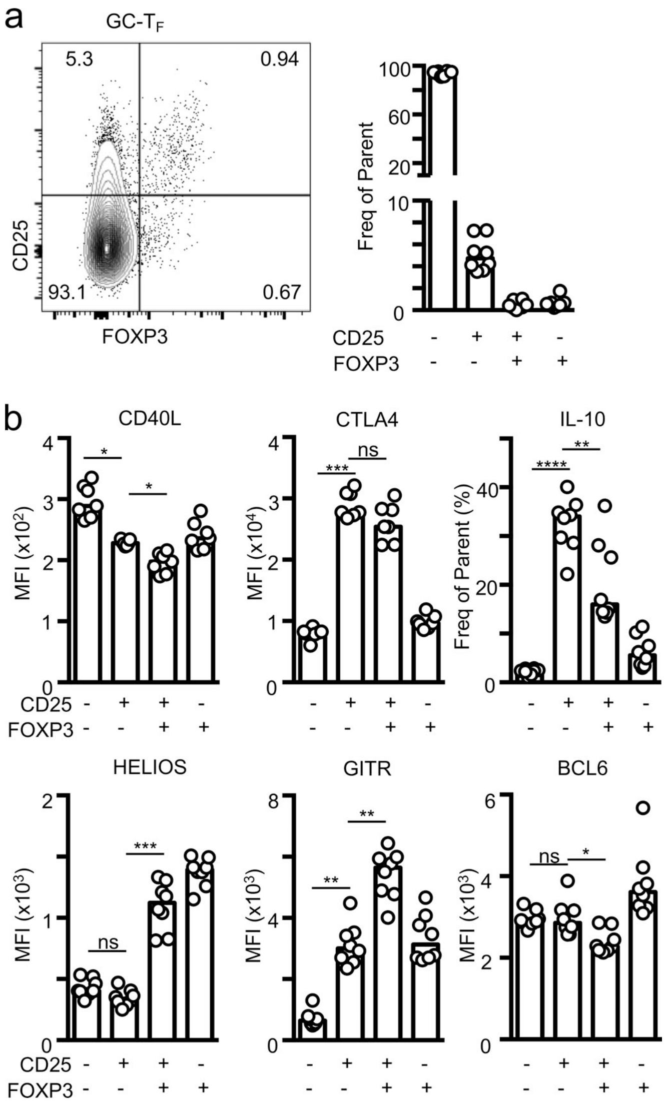

Figure S3. **Phenotypic comparison between CD25⁺FOXP3⁻ and CD25⁺FOXP3⁺ T_F cells. (a)** Flow cytometric plots and quantification (*n* = 12) of the indicated population within tonsillar T_F cells (GC-T_F). Data are representative of three independent experiments. **(b)** Quantification of the indicated protein within the indicated tonsillar cell subset (*n* = 7). Data are representative of two independent experiments. Bars represent medians, and each dot represents a single tonsil donor. ns, not significant; *, P ≤ 0.05; **, P ≤ 0.01; ***, P ≤ 0.001; ****, P ≤ 0.0001, nonparametric Wilcoxon test. MFI, mean fluorescence intensity.

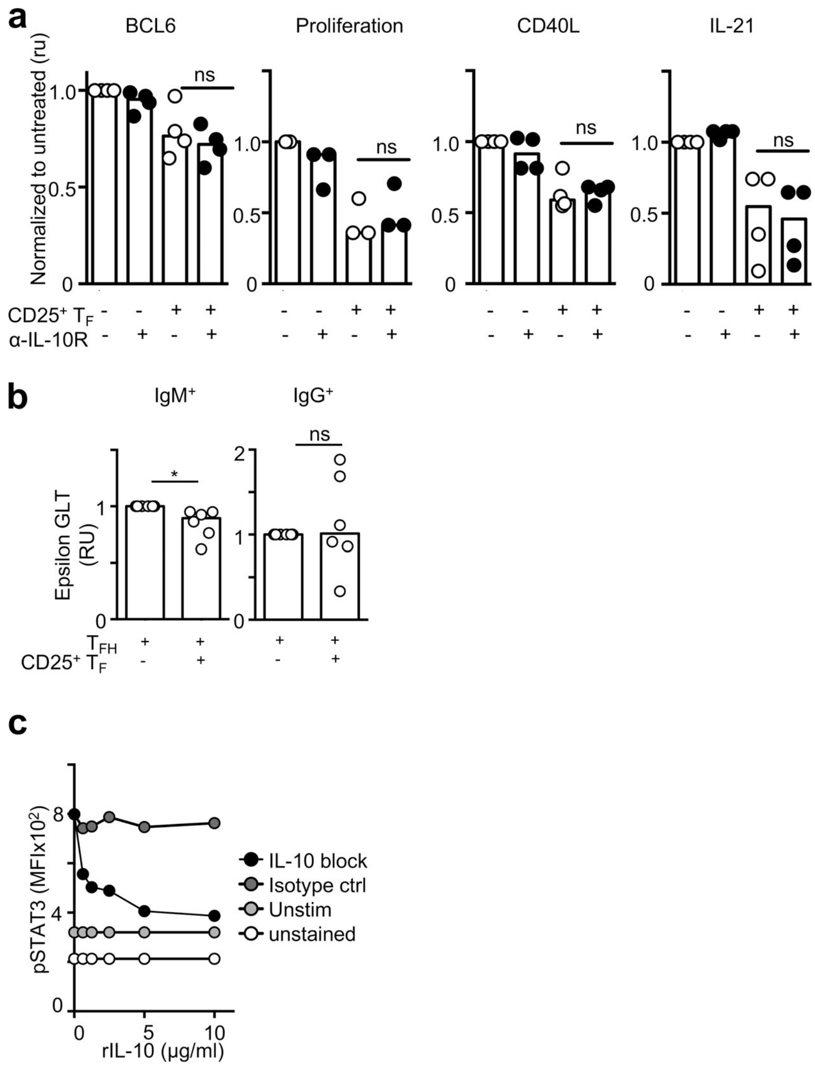

Figure S4. **Repression of T_{FH} cells by CD25⁺ T_F cells is not IL-10 mediated. (a)** Quantification (*n* = 4) of CTV-labeled T_{FH} cells, cocultured with memory B cells with or without CD25⁺ T_F cells, in the presence or absence of an IL-10R blocking antibody (5 µg/ml), showing expression of the indicated proteins after 3 d. Each dot represents a single tonsil donor (*n* = 4). Data were taken from three independent experiments and normalized to T_{FH} cell–only control. ns, not significant, two-tailed Student's *t* test. **(b)** Quantification of εGLTs via qPCR in IgM⁺ or IgG⁺ memory B cells incubated with T_{FH} cells with or without CD25⁺ T_F cells (*n* = 6) for 24 h. GLT expression values were calculated using the ΔΔCT method and were normalized to RPL13A levels, then normalized to the untreated control. Bars represent medians. Data are representative of two independent experiments. ns, not significant; *, P ≤ 0.05. RU, relative units. **(c)** Quantification of pSTAT3 in naive B cells treated with several concentrations of IL-10 for 15 min with or without an IL-10R blocking antibody or an isotype control (Ctrl) antibody (5 µg/ml). Data are representative of two independent experiments.

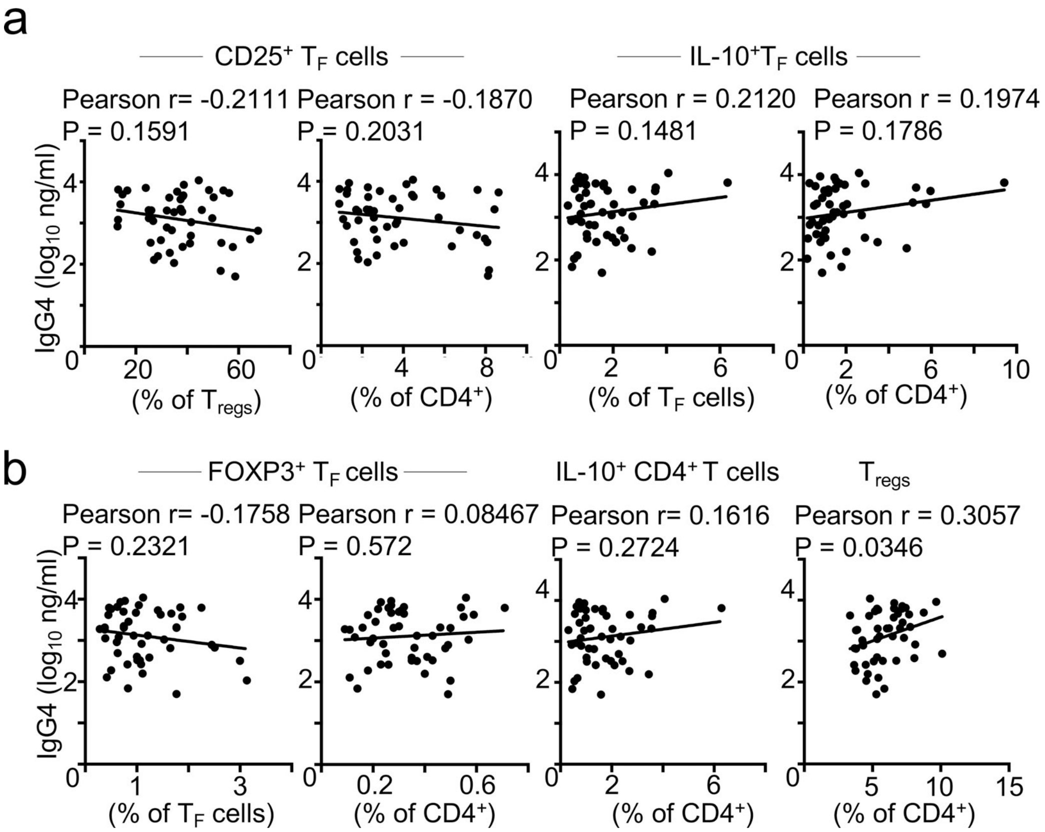

Figure S5. **Tonsillar CD25+ T_F cells do not correlate with total circulating IgG4 levels in the serum. (a and b)** Pearson correlation analyses between serum total IgG4 and the frequency of the indicated cell subset in tonsil (n = 44). Data are representative of two independent experiments that were pooled together.
